# A mutagenic study identifying critical residues for the structure and function of rice manganese transporter OsMTP8.1

**DOI:** 10.1038/srep32073

**Published:** 2016-08-24

**Authors:** Xi Chen, Jiyu Li, Lihua Wang, Gang Ma, Wei Zhang

**Affiliations:** 1 Department of Biochemistry & Molecular Biology, College of Life Sciences, Nanjing Agricultural University, Nanjing, Jiangsu 210095, China

## Abstract

Rice (*Oryza sativa*) MTP8.1 (*Os*MTP8.1) is a tonoplast-localized manganese transporter of the cation diffusion facilitator family. Here we present a structure-function analysis of *Os*MTP8.1 based on the site-directed and random mutagenesis and complementation assays in manganese hypersensitive yeast, in combination with three-dimensional (3D) structure modeling based on the crystal structure of the *Escherichia coli* CDF family member, *Ec*YiiP. Two metal-binding sites are conserved in *Os*MTP8.1 with *Ec*YiiP, one is between transmembrane helices TM2 and TM5, the other is the cytoplasmic C-terminus. In addition to these two metal-binding sites, there may exist other Mn-binding sites such as that at the very end of the CTD. Two residues (R167 and L296) may play an important role for the hinge-like movement of CTDs. Several mutations such as E357A and V374D may affect dimer formation, and S132A may induce a conformational change, resulting in a loss of transport function or modification in metal selectivity. The N-terminus of *Os*MTP8.1 was not functional for Mn transport activity, and the real function of NTD remains to be investigated in the future. The findings of the present study illustrate the structure-function relationship of *Os*MTP8.1 in Mn transport activity, which may also be applied to other plant Mn-CDF proteins.

Manganese (Mn) is an essential micronutrient for plant growth and development. In addition to being a cofactor in many enzymatic processes involved in glucose metabolism and energy production, it also acts as a cofactor of MnSOD in the mitochondrion and as part of the water-splitting complex in PSII in the chloroplast^1^,^2^. Despite its importance, Mn toxicity is common in acidic soils because the amount of exchangeable Mn increases in the soil solution^3^.

Recently, some of the genes responsible for Mn uptake and homeostasis in rice (*Oryza sativa* L.) have been identified. Members of the transporter gene family include natural resistance-associated macrophage protein (NRAMP)^4^,^5^,^6^,^7^, yellowstripe-like (YSL)^8^,^9^, metal tolerance protein (MTP)^10^,^11^, and vacuolar iron transporter (VIT)^12^. Mn uptake in rice roots is mediated by two polarized plasma membrane transporters, *Os*Nramp5 and *Os*MTP9^13^. *Os*Nramp5 is an influx transporter that has been localized to the distal side of the exodermis and endodermis^6^. In contrast, *Os*MTP9 is an efflux transporter that has been localized to the proximal side of both cell layers^11^. *Os*YSL2 and *Os*YSL6 have been implicated in Mn homeostasis. *Os*YSL2 is a metal-nicotianamine transporter that is required for long-distance transport of iron and Mn^8^. *Os*YSL6 translocates Mn from the apoplast to the symplast when plants are exposed to high levels of Mn^9^. *Os*MTP8.1 that was isolated from a rice shoot cDNA library conferred Mn tolerance in *Saccharomyces cerevisiae*, and its expression was found to enhance tolerance and accumulation of Mn, but not other heavy metals. *Os*MTP8.1 has been localized to the tonoplast and transports cytosolic Mn^2+^ into the vacuole. Disruption of *Os*MTP8.1 results in decreased chlorophyll content in the youngest leaf blades. Rice mtp8.1 Tos-17 insertion mutants and transgenic *Os*MTP8.1 RNAi knockdown lines are hypersensitive to Mn and accumulate less Mn in shoots and roots than wild-type plants^10^. However, its structure-function relationship remains elusive to date.

MTPs belong to the cation diffusion facilitator (CDF) transporter family and are important for the maintenance of cation homeostasis in bacteria, yeast, plants, and mammals^14^,^15^,^16^. MTPs play a critical role in removing transition metals from the cytosol either by intracellular sequestration or by cellular export^17^. Phylogenetic analysis has indicated that the CDF family members can be divided into three major groups based on metal ion specificity: Zn-CDF, Fe/Zn-CDF, and Mn-CDF^18^. To date, *Ec*YiiP is the only full-length CDF member that has been crystallized. It was initially characterized at a 3.8 Å resolution^19^ and subsequently improved to 2.9 Å^20^. *Ec*YiiP is a Y-shaped homodimer and each protomer contains three zinc-binding sites. Site A is located at the center of TM2 and TM5 and acts as an active site for zinc transport. This active site is tetrahedrally coordinated by D45 and D49 of TM2 and H153 and D157 of TM5. Site B is located in the intracellular loop that connects TM2 and TM3. Site C hosts four Zn^2+^ ions that mediate an interaction between CTDs and stabilize dimers. The six helices of the transmembrane domain (TMD) are grouped into two bundles with four (TM1–TM2–TM4–TM5) and two (TM3–TM6) helices. When Zn^2+^ binds to site C, the C-terminus moves the TM3-TM6 pair, causing reorientation of TM5, which allosterically changes the geometry of the active Zn^2+^-binding site (site A), and thereby accomplishes transmembrane transport of Zn^2+^.

By using site-directed mutagenesis, Kawachi *et al*. studied the structural basis of Zn^2+^ selectivity and transport activity in *At*MTP1^21^. They found that two Zn-binding sites (sites A and C) are conserved in *At*MTP1 with *Ec*YiiP. They also found that the N-terminus of *At*MTP1 is very important for metal selectivity. They suggested that the N-terminal domain, the His-rich loop, and the leucine zipper motif in TM6 may contribute to the high Zn^2+^ selectivity in *At*MTP1. The His-rich loop of *At*MTP1 acts as a sensor of cytosolic Zn to maintain an essential level of Zn in the cytosol^22^. The sequence characteristics of *Os*MTP8.1 differs from that of *At*MTP1; for example, *Os*MTP8.1 does not have the His-rich loop between TM4 and TM5, and no leucine zipper motif exists within TM6. The sequence of plant Mn-CDF members are highly similar and differ from *Ec*YiiP and *At*MTP1 only in terms of structure-function relationships.

In the present study, we are used site-directed and random mutagenesis to investigate the structural basis of Mn transport activity in a plant Mn-CDF member, *Os*MTP8.1.

## Results

### Subcellular Localization and Metal Selectivity of *Os*MTP8.1

**Figure 1:**
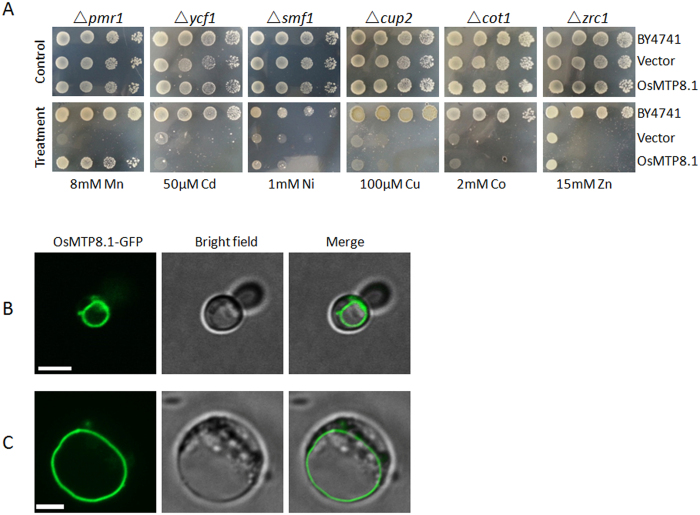
Rice *Os*MTP8.1 is a Mn transporter localized to the vacuolar membrane. (**A**) Dilution series of wild-type and mutant yeast strains transformed with *Os*MTP8.1 or the empty vector were spotted onto plates supplemented with metals as indicated. (**B**) Vacuolar membrane localization of *Os*MTP8.1. C-terminal GFP fusion protein expressed in the *S. cerevisiae* strain Δ*pmr1*. Cells are visualized 24 h after transformation. From left to right, shown are GFP fluorescence, bright-field, and merged images with GFP in green. Scale bar 5 μm. (**C**) Transient expression of *Os*MTP8.1 in rice mesophyll protoplasts. Cells were visualized at 24 h after transformation. From left to right, shown are GFP fluorescence from *Os*MTP8.1-GFP, bright-field, and a merged image. Scale bar 5 μm.

The full-length cDNA fragment of *Os*MTP8.1 (Os03g0226400) was RT-PCR amplified using sequence information derived from the Rice Annotation Project of NCBI (http://www.ncbi.nlm.nih.gov). *Os*MTP8.1 encodes a putative protein of 396 amino acids in length. Phylogenetic analysis of *Os*MTP8.1 indicated that it belongs to the Mn clade of the CDF family^10^. Transport activity of *Os*MTP8.1 was assessed by complementation assay using a number of yeast mutants that were deficient in various metal transporters. *Os*MTP8.1 was expressed in *S. cerevisiae* yeast mutants lacking the endogenous metal transporters for Zn (*zrc1*), Co (*cot1*), Cu (*cup2*), Cd (*ycf1*), Ni (*smf1*), and Mn (*pmr1*). The results showed that a pmr1 strain but not other mutants were restored after transformation with *Os*MTP8.1 (Fig. 1A), thereby suggesting that *Os*MTP8.1 may be a Mn-specific transporter. *Os*MTP8.1 was localized to the vacuolar membrane when it is expressed heterologously in yeast (Fig. 1B). To further confirm the localization of *Os*MTP8.1 in rice, the cDNA of *Os*MTP8.1 was fused in frame to GFP in the expression vector pXZP008. Transient expression of *Os*MTP8.1:GFP in rice mesophyll protoplasts showed that green fluorescence was localized to the tonoplast (Fig. 1C). The tonoplast localization of *Os*MTP8.1 was consistent with the results of a previous study involving onion epidermal cells^10^.

### Site-directed and Random Mutant *Os*MTP8.1 Expression in Yeast from TM1 to TM6

**Figure 2:**
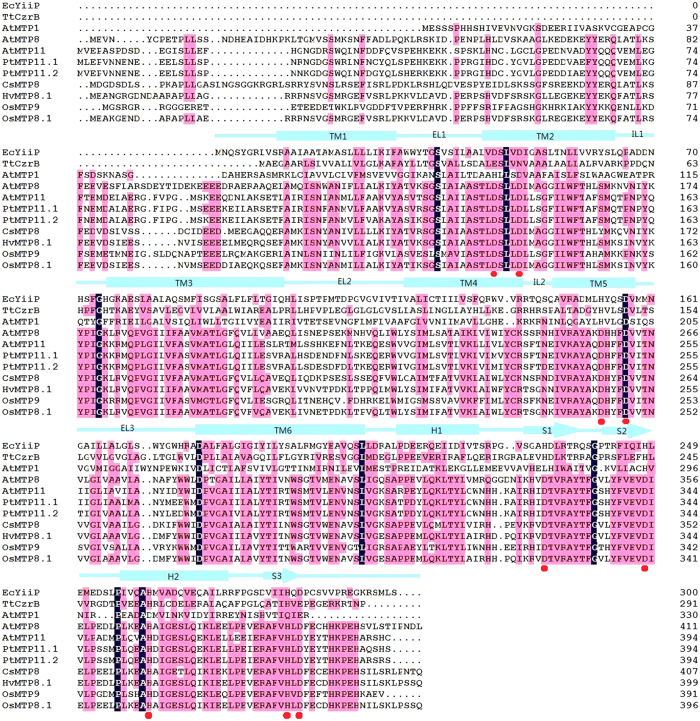
Amino acid sequence alignment of *Os*MTP8.1 with *Ec*YiiP and other CDF family proteins. *Tt*CzrB (GenBank Acc. No. AJ307316.1) from *Thermus thermophilus*, AtMTP1 (GenBank Acc. No. At2g46800), AtMTP8 (GenBank Acc. No. At3g58060) and *At*MTP11 (GenBank Acc. No. At2g39450) from *Arabidopsis thaliana*, *Cs*MTP8 (GenBank Acc. No. AFJ24703) from *Cucumis sativus*, *Hv*MTP8.1 (GenBank Acc. No. AFP33387) from *Hordeum vulgare*, *Pt*MTP11.1 (Phytozome: POPTR_0010s21810) and *Pt*MTP11.2 (Phytozome: POPTR_0008s04940) from *Populus trichocarpa*, *Os*MTP9 (Phytozome: LOC_Os01g03914) and *Os*MTP8.1 (Phytozome: LOC_Os03g12530) from *Oryza sativa*, and *E. coli* K*12 YiiP are aligned.* The sequence of AtMTP1 lacks the histidine-rich loop (D185 to K250). The transmembrane helices (TM), external (vacuolar) loops (EL), intracellular (cytosolic) loops (IL), and C-terminal cytoplasmic a-helices and b-sheets of *Ec*YiiP are shown above the alignment. Residues essential for Mn binding in site A and site C of *Os*MTP8.1 are shown in red. Point denotes a gap in the alignment.

We aligned *Os*MTP8.1 and some other reported Mn-CDF, which included *At*MTP8, *At*MTP11, *Cs*MTP8, *Hv*MTP8.1, *Pt*MTP11.1, *Pt*MTP11.2, and *Os*MTP9 with *Ec*YiiP (Fig. 2). Amino acids that were conserved within Mn-CDF were selected for replacement with aliphatic amino acid Ala. Random mutagenesis was performed on pFL61-OsMTP8.1 by using GeneMorph II random mutagenesis kits (Stratagene) according to the manufacturer’s instructions. The mutation products were transformed into *E. coli*, more than 200 plasmids were extracted. Each plasmid was transformed into the Mn-sensitive mutant *pmr1*, cells were grown for three days on SD-Ura plates containing 6 mM MnCl_2_. The corresponding plasmids from transformed yeast cells which unable to grow on high manganese plates were selected for further study (Fig. S1). Primers for the mutations from random mutagenesis are shown in Table S1. Yeast strain *pmr1* transformed with an empty vector pFL61 was unable to grow on medium supplemented with 3 mM or higher Mn concentrations, but yeast cells expressing pFL61-*Os*MTP8.1 could grow on medium supplemented with up to 8 mM Mn.

**Figure 3:**
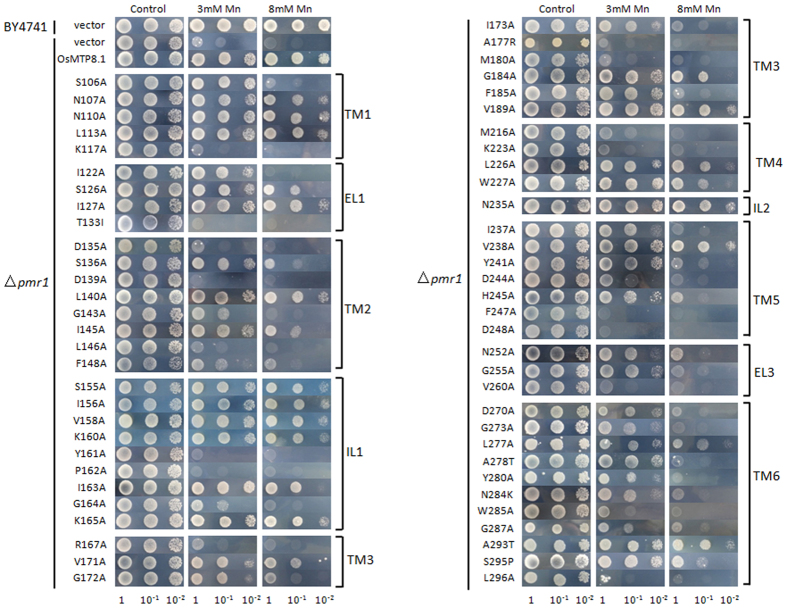
Metal tolerance complementation assays from TM1 to TM6 of *Os*MTP8.1 with random and site-directed mutagenesis. Complementation of *S. cerevisiae* mutant *pmr1* grown on selective media supplemented with 3 mM and 8 mM Mn^2+^. For complementation tests, transformants were pre-cultured in SD-Urea overnight. Pre-cultured cells were diluted to an OD_600 nm_ of 1.0, 10 μL of cell suspensions were spotted on SD-Urea plates supplemented with the indicated concentrations of Mn^2+^. Plates were incubated for 72 h at 30 °C.

Plate growth assays indicated that the mutants have different sensitivities to Mn (Fig. 3). A total of 63 single-substitution mutations conferred nearly the same level of Mn tolerance as that of wild-type *Os*MTP8.1 (Fig. S2). A reduced level of Mn tolerance was conferred by 23 mutations, which included N107A, N110A, L113A, S126A, I127A, L140A, I145A, S155A, I156A, V158A, K160A, I163A, K165A, V171A, G184A, V189A, L226A, W227A, N235A, V238A, L277A, A293T, and S295P. These mutants were able to grow in the presence of 3 mM Mn, but not as well as that of the wild type in the presence of 8 mM Mn. These findings suggest that these amino acids contribute to the activity of *Os*MTP8.1. The other 20 residues possibly play a critical role in the activity of *Os*MTP8.1, as substitution of these amino acids resulted in yeast cells growing only in the presence of 3 mM Mn but not with 8 mM Mn. These residues included S106A, I122A, S136A, G143A, F148A, G164A, G172A, I173A, F185A, I237A, Y241A, H245A, N252A, G255A, D270A, G273A, A278T, Y280A, N284K, and G287A. Finally, 18 mutations that included K117A, T133I, D135A, D139A, L146A, Y161A, P162A, R167A, A177R, M180A, M216A, K223A, D244A, F247A, D248A, V260A, W285A, and L296A resulted in a complete loss of ability to confer Mn tolerance, which indicated that these amino acids are critical for Mn transport activity of OsMTP8.1.

### The function of N- and C-terminal domains of OsMTP8.1

**Figure 4:**
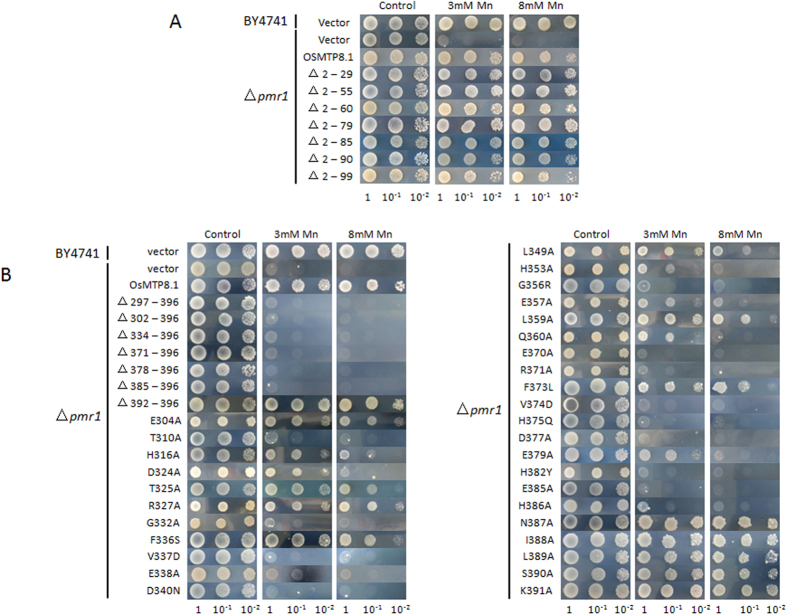
Metal tolerance complementation assays in yeast of N-terminal and C-terminal mutants of *Os*MTP8.1. (**A**) Metal tolerance complementation assays in yeast of N-terminal deletion mutants of *Os*MTP8.1. (**B**) Metal tolerance complementation assays in yeast of C-terminal deletion and site-directed mutagenesis of *Os*MTP8.1. Complementation of *S. cerevisiae* mutant *pmr1* grown on selective media supplemented with 3 mM and 8 mM Mn^2+^. For complementation tests, transformants were pre-cultured in SD-Urea overnight. Pre-cultured cells were diluted to an OD_600 nm_ of 1.0, 10 μL of cell suspensions were spotted on SD-Urea plates supplemented with the indicated concentrations of Mn^2+^. Plates were incubated for 72 h at 30 °C.

The *Os*MTP8.1 protein has six transmembrane (TM) domains, an N-terminal domain, and a C-terminal domain. To study the function of the N- and C-terminal domains of *Os*MTP8.1, we construct several deletion mutants. For the N-terminal domain, Mn tolerance conferred by these deletion mutants was equivalent to that conferred by wild-type *Os*MTP8.1 (Fig. 4A). These results indicate that the N-terminal domain does not contribute to Mn transport activity in *Os*MTP8.1. For the C-terminal domain, we constructed 7 deletion mutants, and only the △392–396 mutant conferred Mn tolerance in the presence of 3 mM and 8 mM Mn, whereas other mutants did not confer any Mn tolerance even at a concentration of 3 mM Mn (Fig. 4B). To further determine which residues are required for Mn transport of *Os*MTP8.1, several substitute mutants were constructed. Mutations E304A, T325A, R327A, F336S, L359A, and F373L conferred reduced levels of Mn tolerance. The ability to confer Mn tolerance was significantly reduced in the H316A, D324A, L349A, E357A, Q360A, and E379A mutants. Approximately 15 mutants showed a complete loss in their ability to confer Mn tolerance; these mutants included T310A, G332A, V337D, E338A, D340N, H353A, G356R, E370A, R371A, V374D, H375Q, D377A, H382Y, E385A, and H386A (Fig. 4B). Mutations conferred nearly the same level of Mn tolerance as wild-type were shown in Fig. S2.

### Zinc tolerance assays

**Figure 5:**
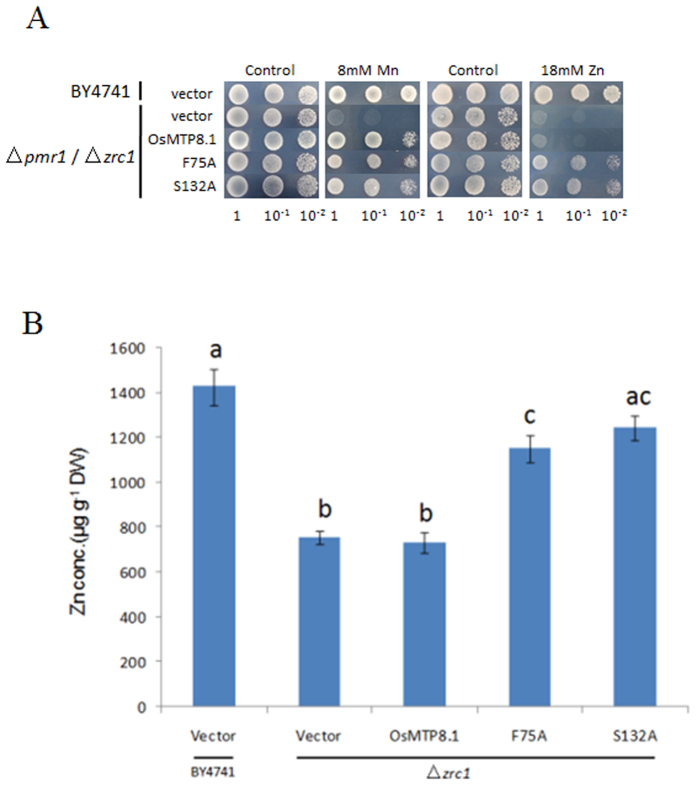
Two mutations of OsMTP8.1 can confer Zn tolerance phenotype in yeast sensitive mutant. (**A**) Yeast cell suspensions of a *zrc1* or *pmr1* transformed with empty vector pFL61, or with pFL61 containing wild-type *Os*MTP8.1 or mutated *Os*MTP8.1 cDNAs were pre-cultured in SD-Urea overnight. Pre-cultured cells were diluted to OD600 nm of 1.0, 10 μL of cell suspensions were spotted on SD-Urea plates supplemented with the indicated concentrations of Mn^2+^or Zn^2+^. Plates were incubated at 30 °C for 72 h. (**B**) Concentration of Zn in yeast cells determined in the wild-type BY4741(WT) and mutant strain zrc1 transformed with empty vector pFL61, OsMTP8.1, and two mutants of OsMTP8.1 (F75A and S132A). Yeast cells were grown in liquid SD medium supplemented with 0.5 mM ZnCl_2_ at an initial OD600 = 0.1. Bars with different letters are significantly different (Tukey’s test, p < 0.01).

The *Os*MTP8.1 mutants were also tested for Zn tolerance in the Zn-hypersensitive yeast strain *zrc1*. The *ZRC1* gene encodes a multicopy suppressor of Zn toxicity that has been localized to the vacuolar membrane of *S. cerevisiae*^23^. Two mutants, F75A and S132A, conferred tolerance to high levels of Zn (18 mM) but not other metals including Cd, Ni, Cu, Co or Fe (Figs 5A and S3). F75 was localized to the NTD, and S132 to EL1. Furthermore, to investigate the metal transport activity of these two mutants, the accumulation of Zn and other metal cations was compared in *zrc1* that expressed OsMTP8.1 or the mutants. The results showed that two mutants accumulated 1.6-fold the amount of Zn of the wild type OsMTP8.1 (Fig. 5B). Significant differences were not detected in the concentrations of other metals (data not shown).

**Figure 6:**
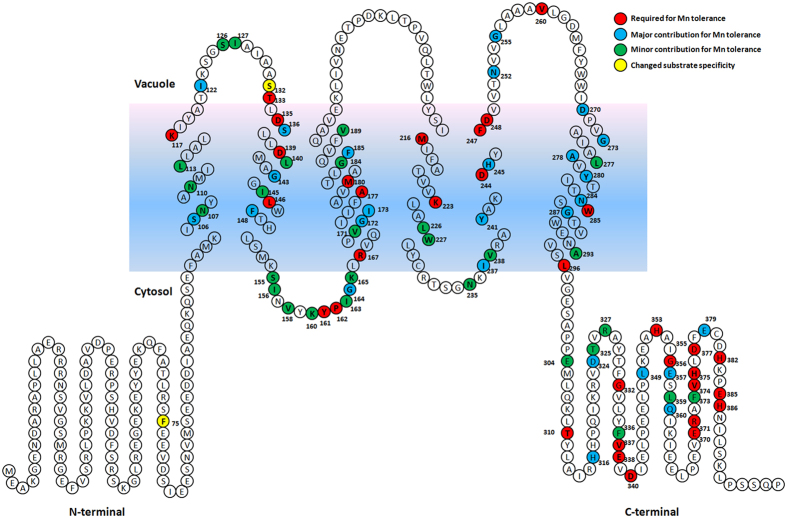
Putative membrane topology model showing residues involved in the ability of *Os*MTP8.1 to confer tolerance to Mn in yeast. The membrane topology model of *Os*MTP8.1 is based on alignment with *Ec*YiiP. Residues whose mutation resulted in loss of Mn tolerance conferred to yeast are shown in red. Residues whose mutation significantly decreased Mn tolerance conferred to yeast are shown in blue. Residues whose mutation slightly decreased Mn tolerance conferred to yeast are shown in green. Residues whose mutation increased the Zn tolerance conferred to yeast are shown in yellow.

Cellular localization of the mutants within yeast cells showed a vacuolar localization similar to the wild-type proteins (Fig. S4), confirming that the mutant proteins are expressed and functional. A summary of effects of all mutations in *Os*MTP8.1 as determined by yeast complementation assays are presented in Fig. 6.

### 3D structural modeling of *Os*MTP8.1

To determine the function of mutated residues involved in the determination of metal transport activity, we constructed a 3D model of *Os*MTP8.1 by using the SWISSMODEL software (Fig. S5). The final models turned out to be strongly biased toward *E. coli* YiiP (3H90). The N-terminal domain of *Os*MTP8.1 was much longer than *Ec*YiiP (Fig. 2) and was not reflected in the 3D model.

Biochemical studies and X-ray structural analysis of *Ec*YiiP indicated that it has three Zn^2+^-binding sites: site A is a tetrahedral Zn^2+^-binding site situated in the transmembrane domain and is composed of two residues from TM2 (Asp45 and Asp49) and two residues from TM5 (His153 and Asp157), two cytoplasmic binding sites, sites B and C. Site B is localized to the intracellular loop that connects TM2 and TM3 (IL1), in which Zn^2+^ is coordinated with one water molecule and three residues Asp68, His71 and His75, and site C in the C-terminal domain that hosts two Zn^2+^ ions bound in a binuclear cluster by Asp285, His232, His248, His283, and His261 from the neighboring subunit of *Ec*YiiP. This site hosts four Zn^2+^ ions that mediate a tight interaction between CTDs and play an important role in stabilizing the dimer^19^,^20^.

**Figure 7:**
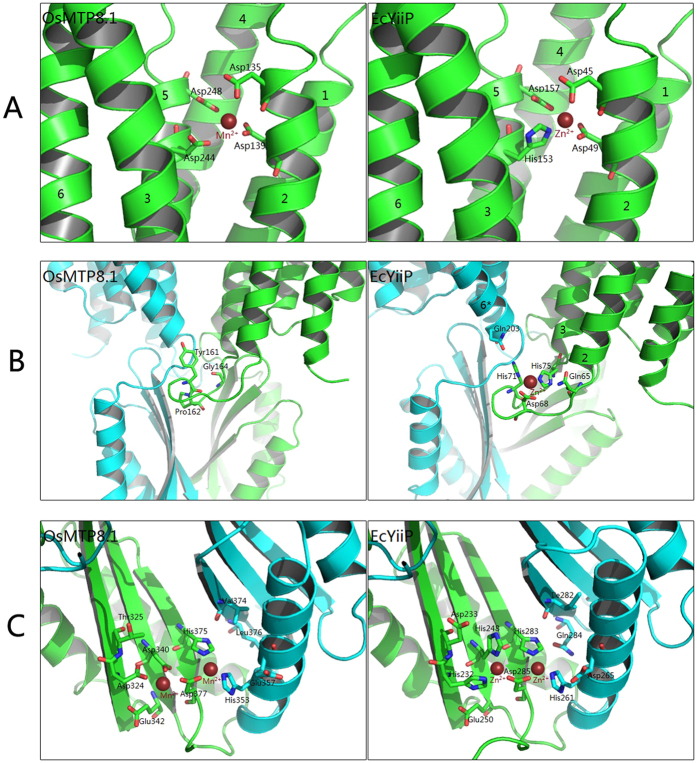
Putative Mn^2+^-binding sites of *Os*MTP8.1. Comparison of putative Mn^2+^-binding sites of *Os*MTP8.1 (left) with characterized Zn^2+^-binding sites of *Ec*YiiP (right). (**A**) Active Mn^2+^-binding site between TM2 and TM5 of *Os*MTP8.1. This site conserved with Zn^2+^-binding site A of *Ec*YiiP. (**B**) Intracellular (cytosolic) loop (IL1) between TM2 and TM3 of *Os*MTP8.1. This region corresponds to Zn^2+^-binding site B of *Ec*YiiP. (**C**) The Mn^2+^-binding site in the C-terminal domain of *Os*MTP8.1, This site conserved with site C of *Ec*YiiP.

According to the 3D model, the active Mn-binding site (site A) of *Os*MTP8.1 consists of D135 and D139 from TM2 and D244 and D248 from TM5 (Fig. 7A). The sequence DxxxD (x = any amino acid) in TM2 and TM5 are conserved in Mn-CDF. Mutations in any of these four residues appeared to abolish Mn transport activity of *Os*MTP8.1. These results suggested functional conservation of site A between *Ec*YiiP and *Os*MTP8.1.

Sequence alignment indicated no conserved regions between *Ec*YiiP and *Os*MTP8.1 in site B (Figs 2 and 7B). However, mutations Y161A and P162A in the intracellular loop between TM2 and TM3 appeared to abolish Mn transport function, and mutation G164A apparently strongly reduced the transport function of OsMTP8.1. These three residues are conserved among Mn-CDFs. These results indicated that IL1 is essential for *Os*MTP8.1 function, but may not likely form a metal-binding site as *Ec*YiiP.

A number of highly conserved residues were detected at site C of *Os*MTP8.1, including D324, D340, H353, H375 and D377, thereby implicating its role in metal binding (Fig. 7C). Mutations D340A, H353A, H375A, and D377A showed a complete loss in the ability to confer Mn tolerance, and D324A presented a significant reduction in function. In addition to the conserved residues, 9 mutations also abolished Mn tolerance, namely, T310A, G332A, V337A, E338A, G356A, E370A, H382A, E385A, and H386A. Sequence alignment indicated that these residues are highly conserved in Mn-CDF but less conserved with *Ec*YiiP, indicating that the structure of CTD for Mn binding in Mn-CDF may be different from that of *Ec*YiiP.

**Figure 8:**
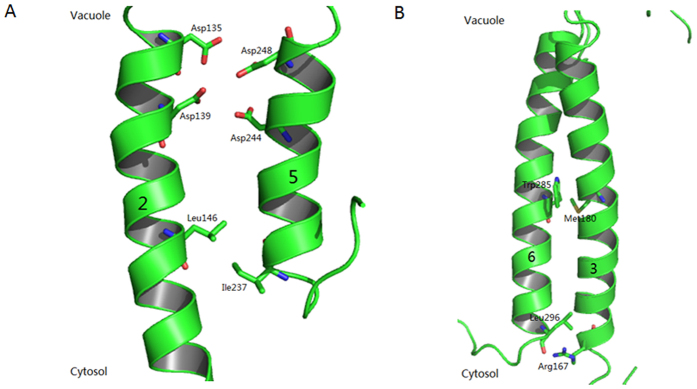
Important residues for the manganese transport activity of OsMTP8.1. (**A**) 3D model view of TM2 and TM5 of *Os*MTP8.1. The active Mn^2+^-binding site is at the vacuolar end between TM2 and TM5. Leu146 and Ile 237 residues are located on the cytosolic side of the active Mn^2+^-binding site. (**B**) 3D model view of TM3-TM6 bundle of *Os*MTP8.1. Met180 and Trp285 residues are localized face to face between TM3 and TM6. Arg167 and Leu296 residues are located on the cytosolic side and near the membrane surface.

Previous biochemical and X-ray analyses have indicated that the *Ec*YiiP homodimer structure is stabilized by an interlocked salt bridge near the membrane surface that is formed by K77 and D207^20^. The corresponding residues for these two residues in *Os*MTP8.1 were R167 and G298, these two residues apparently did not form a salt bridge, and G298A conferred nearly the same level of Mn tolerance as that of wild-type *Os*MTP8.1. Interestingly, we found that R167 and L296 were critical for Mn transport activity of *Os*MTP8.1. These two residues were located near the membrane surface (Fig. 8B); we speculate that they play an important role in hinge movement of *Os*MTP8.1.

## Discussion

According to the phylogenetic study conducted by Montanini *et al*., Mn-CDF proteins can be differentiated from other CDF members by the consensus sequence DXXXD in TM2 and TM5^18^. The active Mn-binding site (site A) of *Os*MTP8.1 is formed by four Asp residues, of which D135 and D139 are located in TM2 and D244 and D248 in TM5 (Fig. 7A). These key residues of the Mn-binding site are highly conserved in other Mn-CDF family proteins (Fig. 2).

In contrast to *Ec*YiiP, *Os*MTP8.1 does not contain the Mn-binding site B in the cytosolic loop (IL1) between TM2 and TM3. This site is also not conserved within other CDF family members such as *Mm*CDF3 from *Maricaulis maris* MCS10^24^ and the plant zinc transporter *At*MTP1^21^. IL1 is the interface for dimerization of *Ec*YiiP protomers^19^. In *Os*MTP8.1, IL1 contains critical residues (Y161, P162, and G164) for transport function (Fig. 7B); these residues may be involved in dimerization of *Os*MTP8.1. These results also suggest that there is an alternative access mechanism for Mn to reach the active transport site from CTD of *Os*MTP8.1 via the intracellular cavity.

The cytoplasmic binding site (site C) of *Os*MTP8.1 has been localized to the CTD-CTD interface and is composed of several highly conserved residues (D324, D340, H353, H375, and D377) (Fig. 7C). *Ec*YiiP showed extensive outer-shell constraints around the binuclear zinc coordination in site C, which were composed of D233, E250, D265, I282, and Q284. These residues apparently establish an extensive network of outer-shell interactions at the CTD interface, thereby stabilizing the CTD-CTD association^20^. In *Os*MTP8.1, these residues correspond to T325, E342, E357, V374, and L376 (Fig. 7C). Yeast complementation assays support the essential role of T325, E357, and V374, and E357 and V374 may contribute to the function of stabilizing the CTD-CTD association in *Os*MTP8.1 (Fig. 7C). The sequence HKPEH from residue 382 to 386 in *Os*MTP8.1 is highly conserved among Mn-CDF and distinguishable from *Ec*YiiP (Fig. 2), and mutations H382A, E385A, and H386A abolished Mn transport activity. We therefore propose that there may be another Mn-binding site at the end of the CTD of *Os*MTP8.1. The 3D model of *Os*MTP8.1 ended at 380 C, and lost the structural information of this site, and the binding information remains to be elucidated in future research investigations. Recently, the 3D structures of the CTDs of several CDF proteins were resolved, e.g., the CzrB of *T. thermophiles*^25^, the apo form of a CDF protein of *T. maritime*^26^, and MamM of *M. gryphiswaldense*^27^. These findings demonstrate that CTDs adopt a metallochaperone-like fold, and the metallochaperones can carry metal ions to various protein targets. It has been suggested that the CTD plays a role in sensing the Mn ions and delivering these to the TMD region of *Os*MTP8.1.

The six transmembrane helices of the TMD in *Ec*YiiP consist of two independent bundles; TM1, TM2, TM4, and TM5 form a four-helix bundle, whereas TM3 and TM6 compose another bundle. The orientation of the TM3–TM6 bundle is stabilized by four interlocking salt bridges formed by K77 of TM3 and D207, which are located in IL3. The charge interlock also plays an important role in stabilizing dimer associations and is essential for Zn transport^20^. When Zn binds to site C, it triggers a hinge-like movement of two CTDs, inter-CTD movements alter the TM3-TM6 bundle orientation and causes reorientation of TM5. The orientation of TM5 with respect to TM2 affects the coordination geometry of the active Zn-binding site, thereby facilitating Zn transport^20^. In *Os*MTP8.1, two residues (R167 from TM3 and L296 from TM6) near the membrane surface play critical roles in Mn transport (Fig. 8B). Our results suggest these two residues may play an important role in the hinge-like movement of CTDs when these bind Mn. Mutation M180A in TM3 and W285A in TM6 abolished Mn transport activity of *Os*MTP8.1 (Fig. 8B). These two hydrophobic residues are localized face-to-face between TM3 and TM6. These mutations may weaken the hydrophobic interactions between TM3 and TM6, thereby resulting in a different orientation for the TM3- TM6 bundle.

In *At*MTP1, Ala-substituted S101 or N258 conferred increased Zn^2+^ tolerance^21^. From the cytosolic side view of the 3D model, these two residues obstruct the entrance to the pore of the active Zn^2+^-binding site between TM2 and TM5. Asn258 is directly adjacent to the His-rich loop and may function as a gate that is controlled by the His-rich loop to control Zn flux. In *Os*MTP8.1, two residues, L146 and I237, were conserved to S101 and N258 of *At*MTP1 (Fig. 8A), and mutant L146A completely lost its ability to transport Mn and I237A conferred reduced levels of Mn tolerance (Fig. 3). There is a long His-rich loop between TM4 and TM5 of *At*MTP1, but the loop between TM4 and TM5 of *Os*MTP8.1 was very short (Fig. 2). Residues L146 and I237 of *Os*MTP8.1 may have different mechanisms for metal transport due to S101 and N258 of *At*MTP1. We propose that mutations L146A and I237A affect the coordination geometry between TM2 and TM5 and abolish Mn transport activity in *Os*MTP8.1.

The N-terminal deletions showed no effect of Mn transport activity compared to that in wild-type *Os*MTP8.1 (Fig. 4A). In *At*MTP1 and *Ptd*MTP1, two Cys residues are required for Zn transport activity; these two residues are well conserved among plant Zn-CDF proteins^21^,^28^. The N-terminal deletion mutant, which lacks the Cys residues, also did not confer Zn tolerance. Compared to the Zn-CDF proteins, the function of the N-terminal domain of *Os*MTP8.1 requires further investigation.

Previous studies have shown that several sites influence metal specificity in CDF family proteins. In *At*MTP1, the His-rich loop of IL2 and sequences within NTD are responsible for metal selectivity, and several residues within transmembrane domain were also found to influence metal selectivity^21^. In *Os*MTP1, residue L82, which is located in EL1, may also play a role in substrate specificity^29^. This also found in other CDF family proteins such as *Sc*ZRC1^30^. Two mutants, F75A located in the NTD and S132A located in EL1, also conferred tolerance to Zn besides Mn.

In conclusion, by using site-directed and random mutagenesis and complementation assays in yeast, and in combination with 3D structure modeling based on the *E. coli* Zn transporter YiiP, we have identified residues that are essential for Mn transport in the plant Mn-CDF member, *Os*MTP8.1. We also presented a structure-function relationship involving *Os*MTP8.1 for Mn transport activity that may also be applied to other plant Mn-CDF proteins. Two metal binding sites are conserved in *Os*MTP8.1 with *Ec*YiiP, one is the active Mn-binding site between TM2 and TM5, and the other is the CTD-binding site. In addition, there may also be other Mn binding sites such as the site at the very end of CTD. Several mutations may affect dimer formation or cause a conformational change in the protein, thereby resulting in a loss of transport function or modification in metal selectivity. The N-terminus of *Os*MTP8.1 is not functional for Mn transport activity, and the real function of NTD remains to be investigated in future studies.

## Methods

### Site-directed and random mutagenesis of *Os*MTP8.1

*Os*MTP8.1 was inserted into the *Not*I sites of pFL61^31^ to obtain the expression plasmid. Site-directed mutagenesis and random mutagenesis were performed on the pFL61-*Os*MTP8.1 vector using QuikChange II XL site-directed mutagenesis and GeneMorph II random mutagenesis kits (Stratagene) according to the manufacturer’s instructions^32^. Sequences encoding N- or C-terminally truncated mutants of OsMTP8.1 were amplified by PCR, and the DNA fragments obtained were inserted into the *Not*I site of the pFL61 vector. The primers used and the mutations induced are listed in Table S2. All constructs generated in the present study were verified by DNA sequencing.

### Heterologous expression of mutated OsMTP8.1 in yeast

The mutants and the wild-type yeast strains used in this research were obtained from the Euroscarf collection^33^. Plasmids were transformed into the Mn-sensitive mutant Y04534 (*pmr1*) or Zn-sensitive mutant Y00829 (*zrc1*) and its parental strain BY4741 for Mn and Zn complementation analyses. Yeast transformation was performed by using the lithium acetate/PEG transformation method^34^. Positive colonies were selected on synthetic dropout (SD) plates containing the appropriate selective markers. Yeast strains expressing empty vector, wild-type *Os*MTP8.1, or mutated OsMTP8.1 variants were pre-cultured in SD-Ura liquid medium at 30 °C for 16 h. Pre-cultured cells were diluted to an OD_600_ nm of 1.0, and 10-μL aliquots were spotted onto SD-Ura plates containing various concentrations of metals as indicated elsewhere^35^. Plates were incubated at 30 °C for 3 days.

### Subcellular localization in rice protoplast and yeast

For the subcellular localization in rice protoplasts, the *Os*MTP8.1 cDNA fragment without a stop codon was amplified and was cloned into the *BamH*I and *Kpn*I sites of pXZP008^36^. The construct was transformed into rice mesophyll protoplasts and GFP fluorescence was observed using a confocal laser scanning microscopy (Confocal System-UitraView VOX, Perkin Elmer). For the subcellular localization in yeast, the ORFs were amplified by using primers containing the *Mlu*I site, and cloned into the yeast expression vector pFL61-GFP. The construct was transformed into *pmr1* and GFP signal was observed by confocal laser scanning microscopy.

### Bioinformatics analysis of OsMTP8.1

Multiple sequence alignments of TtCzrB (GenBank Acc. No. AJ307316.1) from Thermus thermophilus, *Arabidopsis thaliana* AtMTP1 (GenBank Acc. No. At2g46800), *At*MTP8 (GenBank Accession Number At3g58060) and *At*MTP11 (GenBank Acc. No. At2g39450), *Cucumis sativus Cs*MTP8 (GenBank Acc. No. AFJ24703), *Hordeum vulgare Hv*MTP8.1 (GenBank Acc. No. AFP33387), *Pt*MTP11.1 (Phytozome: POPTR_0010s21810) and *Pt*MTP11.2 (Phytozome: POPTR_0008s04940) from *Populus trichocarpa*, *Os*MTP9 (Phytozome: LOC_Os01g03914) and *Os*MTP8.1 (Phytozome: LOC_Os03g12530) from *Oryza sativa,* and *E. coli* K12 YiiP (P69380) were performed using multalin^37^. The 3D model of OsMTP8.1 was generated by homology modeling using the SWISSMODEL software (http://swissmodel.expasy.org/)^38^,^39^,^40^,^41^ based on the structure of *Ec*YiiP (PDB ID 3H90). Images were generated by using PyMOL 1.6.x.

## Additional Information

**How to cite this article**: Chen, X. *et al*. A mutagenic study identifying critical residues for the structure and function of rice manganese transporter OsMTP8.1. *Sci. Rep.* **6**, 32073; doi: 10.1038/srep32073 (2016).

## Supplementary Material

Supplementary Information

## References

[b1] Pittman J. K. Managing the manganese: molecular mechanisms of manganese transport and homeostasis. New Phytol 167, 733–742 (2005). 16101910 10.1111/j.1469-8137.2005.01453.x

[b2] Peiter E. . A secretory pathway-localized cation diffusion facilitator confers plant manganese tolerance. Proc Natl Acad Sci USA 104, 8532–8537 (2007). 17494768 10.1073/pnas.0609507104 PMC1895984

[b3] Marschner H. & Marschner P. Marschner’s mineral nutrition of higher plants. 3rd edn (Elsevier/Academic Press, 2012).

[b4] Yang M. . OsNRAMP5 contributes to manganese translocation and distribution in rice shoots. J Exp Bot 65, 4849–4861 (2014). 24963001 10.1093/jxb/eru259 PMC4144776

[b5] Yang M. . OsNRAMP3 is a vascular bundles-specific manganese transporter that is responsible for manganese distribution in rice. Plos One 8, e83990 (2013). 24391861 10.1371/journal.pone.0083990 PMC3877151

[b6] Sasaki A., Yamaji N., Yokosho K. & Ma J. F. Nramp5 is a major transporter responsible for manganese and cadmium uptake in rice. Plant Cell 24, 2155–2167 (2012). 22589467 10.1105/tpc.112.096925 PMC3442593

[b7] Ishimaru Y. . Characterizing the role of rice NRAMP5 in Manganese, Iron and Cadmium Transport. Sci Rep 2, 286 (2012). 22368778 10.1038/srep00286 PMC3285952

[b8] Ishimaru Y. . Rice metal-nicotianamine transporter, OsYSL2, is required for the long-distance transport of iron and manganese. Plant J 62, 379–390 (2010). 20128878 10.1111/j.1365-313X.2010.04158.x

[b9] Sasaki A., Yamaji N., Xia J. & Ma J. F. OsYSL6 is involved in the detoxification of excess manganese in rice. Plant Physiol 157, 1832–1840 (2011). 21969384 10.1104/pp.111.186031 PMC3327210

[b10] Chen Z. . Mn tolerance in rice is mediated by MTP8.1, a member of the cation diffusion facilitator family. J Exp Bot 64, 4375–4387 (2013). 23963678 10.1093/jxb/ert243 PMC3808320

[b11] Ueno D. . A polarly localized transporter for efficient manganese uptake in rice. Nat Plants 1, doi: 10.1038/nplants.2015.170 (2015). 27251715

[b12] Zhang Y., Xu Y. H., Yi H. Y. & Gong J. M. Vacuolar membrane transporters OsVIT1 and OsVIT2 modulate iron translocation between flag leaves and seeds in rice. Plant J 72, 400–410 (2012). 22731699 10.1111/j.1365-313X.2012.05088.x

[b13] Sasaki A., Yamaji N. & Ma J. F. Transporters involved in mineral nutrient uptake in rice. J Exp Bot 67, 3645–3653 (2016). 26931170 10.1093/jxb/erw060

[b14] Nies D. H. Efflux-mediated heavy metal resistance in prokaryotes. FEMS Microbiol Rev 27, 313–339 (2003). 12829273 10.1016/S0168-6445(03)00048-2

[b15] Maser P. . Phylogenetic relationships within cation transporter families of Arabidopsis. Plant Physiol 126, 1646–1667 (2001). 11500563 10.1104/pp.126.4.1646 PMC117164

[b16] Kramer U., Talke I. N. & Hanikenne M. Transition metal transport. FEBS Lett 581, 2263–2272 (2007). 17462635 10.1016/j.febslet.2007.04.010

[b17] Gustin J. L., Zanis M. J. & Salt D. E. Structure and evolution of the plant cation diffusion facilitator family of ion transporters. BMC Evol Biol 11, 76 (2011). 21435223 10.1186/1471-2148-11-76 PMC3073911

[b18] Montanini B., Blaudez D., Jeandroz S., Sanders D. & Chalot M. Phylogenetic and functional analysis of the Cation Diffusion Facilitator (CDF) family: improved signature and prediction of substrate specificity. BMC Genomics 8, 107 (2007). 17448255 10.1186/1471-2164-8-107 PMC1868760

[b19] Lu M. & Fu D. Structure of the zinc transporter YiiP. Science 317, 1746–1748 (2007). 17717154 10.1126/science.1143748

[b20] Lu M., Chai J. & Fu D. Structural basis for autoregulation of the zinc transporter YiiP. Nat Struct Mol Biol 16, 1063–1067 (2009). 19749753 10.1038/nsmb.1662 PMC2758918

[b21] Kawachi M. . Amino acid screening based on structural modeling identifies critical residues for the function, ion selectivity and structure of Arabidopsis MTP1. FEBS J 279, 2339–2356 (2012). 22520078 10.1111/j.1742-4658.2012.08613.x

[b22] Tanaka N. . Characterization of the histidine-rich loop of Arabidopsis vacuolar membrane zinc transporter AtMTP1 as a sensor of zinc level in the cytosol. Plant Cell Physiol 56, 510–519 (2015). 25516571 10.1093/pcp/pcu194

[b23] Miyabe S., Izawa S. & Inoue Y. The Zrc1 is involved in zinc transport system between vacuole and cytosol in Saccharomyces cerevisiae. Biochem Biophys Res Commun 282, 79–83 (2001). 11263974 10.1006/bbrc.2001.4522

[b24] Russell D. & Soulimane T. Evidence for zinc and cadmium binding in a CDF transporter lacking the cytoplasmic domain. Febs Letters 586, 4332–4338 (2012). 23127559 10.1016/j.febslet.2012.10.043

[b25] Cherezov V. . Insights into the mode of action of a putative zinc transporter CzrB in Thermus thermophilus. Structure 16, 1378–1388 (2008). 18786400 10.1016/j.str.2008.05.014 PMC2614558

[b26] Higuchi T., Hattori M., Tanaka Y., Ishitani R. & Nureki O. Crystal structure of the cytosolic domain of the cation diffusion facilitator family protein. Proteins 76, 768–771 (2009). 19422055 10.1002/prot.22444

[b27] Zeytuni N. . Cation diffusion facilitators transport initiation and regulation is mediated by cation induced conformational changes of the cytoplasmic domain. Plos One 9, e92141 (2014). 24658343 10.1371/journal.pone.0092141 PMC3962391

[b28] Blaudez D., Kohler A., Martin F., Sanders D. & Chalot M. Poplar metal tolerance protein 1 confers zinc tolerance and is an oligomeric vacuolar zinc transporter with an essential leucine zipper motif. Plant Cell 15, 2911–2928 (2003). 14630973 10.1105/tpc.017541 PMC282827

[b29] Menguer P. K. . Functional analysis of the rice vacuolar zinc transporter OsMTP1. J Exp Bot 64, 2871–2883 (2013). 23761487 10.1093/jxb/ert136 PMC3697945

[b30] Lin H. . Gain-of-function mutations identify amino acids within transmembrane domains of the yeast vacuolar transporter Zrc1 that determine metal specificity. Biochem J 422, 273–283 (2009). 19538181 10.1042/BJ20090853 PMC4378827

[b31] Minet M., Dufour M. E. & Lacroute F. Complementation of Saccharomyces cerevisiae auxotrophic mutants by Arabidopsis thaliana cDNAs. Plant J 2, 417–422 (1992). 1303803 10.1111/j.1365-313x.1992.00417.x

[b32] Podar D. . Metal Selectivity Determinants in a Family of Transition Metal Transporters. Journal of Biological Chemistry 287, 3185–3196 (2012). 22139846 10.1074/jbc.M111.305649 PMC3270973

[b33] Winzeler E. A. . Functional characterization of the S. cerevisiae genome by gene deletion and parallel analysis. Science 285, 901–906 (1999). 10436161 10.1126/science.285.5429.901

[b34] Gietz R. D., Schiestl R. H., Willems A. R. & Woods R. A. Studies on the transformation of intact yeast cells by the LiAc/SS-DNA/PEG procedure. Yeast 11, 355–360 (1995). 7785336 10.1002/yea.320110408

[b35] Peiter E., Fischer M., Sidaway K., Roberts S. K. & Sanders D. The Saccharomyces cerevisiae Ca2+ channel Cch1pMid1p is essential for tolerance to cold stress and iron toxicity. FEBS Lett 579, 5697–5703 (2005). 16223494 10.1016/j.febslet.2005.09.058

[b36] Shi B. . OsDMI3 is a novel component of abscisic acid signaling in the induction of antioxidant defense in leaves of rice. Mol Plant 5, 1359–1374 (2012). 22869603 10.1093/mp/sss068

[b37] Corpet F. Multiple sequence alignment with hierarchical clustering. Nucleic Acids Res 16, 10881–10890 (1988). 2849754 10.1093/nar/16.22.10881 PMC338945

[b38] Arnold K., Bordoli L., Kopp J. & Schwede T. The SWISS-MODEL workspace: a web-based environment for protein structure homology modelling. Bioinformatics 22, 195–201 (2006). 16301204 10.1093/bioinformatics/bti770

[b39] Kiefer F., Arnold K., Kunzli M., Bordoli L. & Schwede T. The SWISS-MODEL Repository and associated resources. Nucleic Acids Res 37, D387–D392 (2009). 18931379 10.1093/nar/gkn750 PMC2686475

[b40] Guex N., Peitsch M. C. & Schwede T. Automated comparative protein structure modeling with SWISS-MODEL and Swiss-PdbViewer: a historical perspective. Electrophoresis 30 Suppl 1, S162–S173 (2009). 19517507 10.1002/elps.200900140

[b41] Biasini M. . SWISS-MODEL: modelling protein tertiary and quaternary structure using evolutionary information. Nucleic Acids Res 42, W252–W258 (2014). 24782522 10.1093/nar/gku340 PMC4086089

