# Supplementary material for: A mutagenic study identifying critical residues for the structure and function of rice manganese transporter OsMTP8.1
Source: Sci Rep. 2016 Aug 24;6:32073. doi: 10.1038/srep32073 (PMC4995437; doi:10.1038/srep32073)
Supplement: Supplementary Information [file srep32073-s1.doc]

**A mutagenic study identifying critical residues for the structure and function of rice manganese transporter OsMTP8.1**

Xi Chen^#^, Jiyu Li^#^, Lihua Wang, Gang Ma, Wei Zhang

Department of Biochemistry & Molecular Biology, College of Life Sciences, Nanjing Agricultural University, Nanjing, Jiangsu 210095, China

^#^ Xi Chen and Jiyu Li contributed equally to this work.

**Corresponding author:**

Wei Zhang, Department of Biochemistry & Molecular Biology, College of Life Sciences, Nanjing Agricultural University, Nanjing, Jiangsu 210095, China.

Tel: +86 25 84396556

Fax: +86 25 84396671

E-mail: [wzhang@njau.edu.cn](mailto:wzhang@njau.edu.cn)

**Supplementary material**

Table S1. List of primers used for cloning mutations of OsMTP8.1 from random mutagenesis, mutation sites are underlined.

|  | Sequence of forward primer | Sequence of reverse primer |
| --- | --- | --- |
| T133I  W267C  A278T  I282V  N284K  S295P  T310R  F336S  V337D  D340N  G356R  P367T  F373L  V374D  H375Q  H382Y | 5’-CTATAGCTGCCTCGATATTGGACTCGCTGCTTG-3’  5’-GGTGATATGTTCTACTGCTGGATTGACCCAGTTG-3’  5’-GGTGCAATTGCCCTTACAGTGTACACAATTACAAAC-3’  5’-CTTGCAGTGTACACAGTTACAAACTGGTCTGGAAC-3’  5’-CAGTGTACACAATTACAAAGTGGTCTGGAACGGTGTG-3’  5’-GTGTGGGAAAATGCAGTGCCACTAGTGGGTGAATCAGC-3’  5’-ATGCTGCAGAAGTTGAGATATTTGGCCATCAGAC-3’  5’-CTTTGGGGTTCTTTACTCTGTTGAGGTTGACATTG-3’  5’-CTTTGGGGTTCTTTACTTTGATGAGGTTGACATTGAGC-3’  5’-CTTTACTTTGTTGAGGTTAACATTGAGCTGCCAGAG-3’  5’-GAGGCACATGCTATTCGAGAATCACTTCAAATAAAG-3’  5’-ATAAAGATCGAGGAGCTCACAGAAGTCGAGCGGGCAT-3’  5’-AGAAGTCGAGCGGGCACTTGTTCACCTTGATTTCG-3’  5’-AGTCGAGCGGGCATTTGATCACCTTGATTTCGAGT-3’  5’-CGAGCGGGCATTTGTTCAACTTGATTTCGAGTGTG-3’  5’-GATTTCGAGTGTGACTACAAGCCAGAGCACAAC-3’ | 5’-CAAGCAGCGAGTCCAATATCGAGGCAGCTATAG-3’  5’-CAACTGGGTCAATCCAGCAGTAGAACATATCACC-3’  5’-GTTTGTAATTGTGTACACTGTAAGGGCAATTGCACC-3’  5’-GTTCCAGACCAGTTTGTAACTGTGTACACTGCAAG-3’  5’-CACACCGTTCCAGACCACTTTGTAATTGTGTACACTG-3’  5’-GCTGATTCACCCACTAGTGGCACTGCATTTTCCCACAC-3’  5’-GTCTGATGGCCAAATATCTCAACTTCTGCAGCAT-3’  5’-CAATGTCAACCTCAACAGAGTAAAGAACCCCAAAG-3’  5’-GCTCAATGTCAACCTCATCAAAGTAAAGAACCCCAAAG-3’  5’-CTCTGGCAGCTCAATGTTAACCTCAACAAAGTAAAG-3’  5’-CTTTATTTGAAGTGATTCTCGAATAGCATGTGCCTC-3’  5’-ATGCCCGCTCGACTTCTGTGAGCTCCTCGATCTTTAT-3’  5’-CGAAATCAAGGTGAACAAGTGCCCGCTCGACTTCT-3’  5’-ACTCGAAATCAAGGTGATCAAATGCCCGCTCGACT-3’  5’-CACACTCGAAATCAAGTTGAACAAATGCCCGCTCG-3’  5’-GTTGTGCTCTGGCTTGTGTCACACTCGAAATC-3’ |

Table S2. List of primers used for cloning mutant OsMTP8.1 cDNAs by using site directed mutation, mutation sites are underlined.

|  | Sequence of forward primer | Sequence of reverse primer |
| --- | --- | --- |
| F29A  G56A  Y64A  Q68A  F75A  E77A  E83A  E90A  D91A  K104A  I105A  S106A  N107A  N110A  L113A  L114A  K117A  I122A  K123A  S126A  I127A  S132A | 5’-TCGATGCGCGGGGAGGCCGTGTCGCGGCTGCCCAAG-3’  5’-CTTCTCCCGCTCCAAGGCCCTCCGCGAAGGAGAG-3’  5’-CGAAGGAGAGAAAGAAGCCTATGAGAAACAATTTG-3’  5’-GAATACTATGAGAAAGCATTTGCCACCTTGAGATC-3’  5’-GCTCACCTTGAGATCCGCTGAGGAAGTTGATTCAATAG-3’  5’-CCTTGAGATCCTTTGAGGCGGTTGATTCAATAGAAG-3’  5’-AGTTGATTCAATAGAAGCATCCAATGTAATGAGTG-3’  5’-CAATGTAATGAGTGAAGCGGATGATATTGCGGAG-3’  5’-ATGTAATGAGTGAAGAAGCTGATATTGCGGAGCAAAAG-3’  5’-GAGTGAGTTTGCCATGGCGATATCAAACTATGCAAAC-3’  5’-GAGTTTGCCATGAAGGCATCAAACTATGCAAACATG-3’  5’-GTTTGCCATGAAGATAGCAAACTATGCAAACATGATTC-3’  5’-TGCCATGAAGATATCAGCCTATGCAAACATGATTC-3’  5’-GAAGATATCAAACTATGCAGCCATGATTCTTTTGGCGTTG-3’  5’-CTATGCAAACATGATTGCTTTGGCGTTGAAGATATATG-3’  5’-TGCAAACATGATTCTTGCGGCGTTGAAGATATATG-3’  5’-GATTCTTTTGGCGTTGGCGATATATGCTACAATTAAAAG-3’  5’-GAAGATATATGCTACAGCTAAAAGTGGATCAATTGCT-3’  5’-GATATATGCTACAATTGCAAGTGGATCAATTGCTAT-3’  5’-CTACAATTAAAAGTGGAGCAATTGCTATAGCTGCCTC-3’  5’-CAATTAAAAGTGGATCAGCTGCTATAGCTGCCTCGAC-3’  5’-CAATTGCTATAGCTGCCGCGACATTGGACTCGCTGC-3’ | 5’-TTGGGCAGCCGCGACACGGCCTCCCCGCGCATCGAC-3’  5’-CTCTCCTTCGCGGAGGGCCTTGGAGCGGGAGAAG-3’  5’-CAAATTGTTTCTCATAGGCTTCTTTCTCTCCTTCG-3’  5’-GATCTCAAGGTGGCAAATGCTTTCTCATAGTATTC-3’  5’-CTATTGAATCAACTTCCTCAGCGGATCTCAAGGTGGCA-3’  5’-CTTCTATTGAATCAACCGCCTCAAAGGATCTCAAGG-3’  5’-CACTCATTACATTGGATGCTTCTATTGAATCAACT-3’  5’-CTCCGCAATATCATCCGCTTCACTCATTACATTG-3’  5’-CTTTTGCTCCGCAATATCAGCTTCTTCACTCATTACAT-3’  5’-GTTTGCATAGTTTGATATCGCCATGGCAAACTCACTC-3’  5’-CATGTTTGCATAGTTTGATGCCTTCATGGCAAACTC-3’  5’-GAATCATGTTTGCATAGTTTGCTATCTTCATGGCAAAC-3’  5’-GAATCATGTTTGCATAGGCTGATATCTTCATGGCA-3’  5’-CAACGCCAAAAGAATCATGGCTGCATAGTTTGATATCTTC-3’  5’-CATATATCTTCAACGCCAAAGCAATCATGTTTGCATAG-3’  5’-CATATATCTTCAACGCCGCAAGAATCATGTTTGCA-3’  5’-CTTTTAATTGTAGCATATATCGCCAACGCCAAAAGAATC-3’  5’-AGCAATTGATCCACTTTTAGCTGTAGCATATATCTTC-3’  5’-ATAGCAATTGATCCACTTGCAATTGTAGCATATATC-3’  5’-GAGGCAGCTATAGCAATTGCTCCACTTTTAATTGTAG-3’  5’-GTCGAGGCAGCTATAGCAGCTGATCCACTTTTAATTG-3’  5’-GCAGCGAGTCCAATGTCGCGGCAGCTATAGCAATTG-3’ |

|  | Sequence of forward primer | Sequence of reverse primer |
| --- | --- | --- |
| L134A  D135A  S136A  L137A  L138A  D139A  L140A  M141A  A142T  G143A  G144A  I145A  L146A  F148A  T149A  H150A  L151A  S155A  I156A  N157A  V158A  Y159A  K160A  Y161A  P162A | 5’-CTATAGCTGCCTCGACAGCGGACTCGCTGCTTGATC-3’  5’-GCTGCCTCGACATTGGCCTCGCTGCTTGATCTC-3’  5’-CTGCCTCGACATTGGACGCGCTGCTTGATCTCATG-3’  5’-CTCGACATTGGACTCGGCGCTTGATCTCATGGCTGGTG-3’  5’-CGACATTGGACTCGCTGGCTGATCTCATGGCTGGTGG-3’  5’-CATTGGACTCGCTGCTTGCTCTCATGGCTGGTGGTATC-3’  5’-GGACTCGCTGCTTGATGCCATGGCTGGTGGTATCCT-3’  5’-CTCGCTGCTTGATCTCGCGGCTGGTGGTATCCTTTG-3’  5’-GCTGCTTGATCTCATGACTGGTGGTATCCTTTGGT-3’  5’-CTTGATCTCATGGCTGCTGGTATCCTTTGGTTC-3’  5’-GATCTCATGGCTGGTGCTATCCTTTGGTTCACAC-3’  5’-TCTCATGGCTGGTGGTGCCCTTTGGTTCACACATCT-3’  5’-CATGGCTGGTGGTATCGCTTGGTTCACACATCTCTC-3’  5’-CTGGTGGTATCCTTTGGGCCACACATCTCTCGATGAAG-3’  5’-GTGGTATCCTTTGGTTCGCACATCTCTCGATGAAGAG-3’  5’-GTATCCTTTGGTTCACAGCTCTCTCGATGAAGAGCAT-3’  5’-CTTTGGTTCACACATGCCTCGATGAAGAGCATC-3’  5’-ACATCTCTCGATGAAGGGCATCAACGTCTACAAGT-3’  5’-ATCTCTCGATGAAGAGCGCCAACGTCTACAAGTAT-3’  5’-CTCGATGAAGAGCATCGCCGTCTACAAGTATCCT-3’  5’-CGATGAAGAGCATCAACGCCTACAAGTATCCTATTG-3’  5’-GAAGAGCATCAACGTCGCCAAGTATCCTATTGGT -3’  5’-AGAGCATCAACGTCTACGCGTATCCTATTGGTAAAC-3’  5’-CATCAACGTCTACAAGGCTCCTATTGGTAAACTGAG-3’  5’-CAACGTCTACAAGTATGCTATTGGTAAACTGAGGGT-3’ | 5’-GATCAAGCAGCGAGTCCGCTGTCGAGGCAGCTATAG-3’  5’-GAGATCAAGCAGCGAGGCCAATGTCGAGGCAGC-3’  5’-CATGAGATCAAGCAGCGCGTCCAATGTCGAGGCAG-3’  5’-CACCAGCCATGAGATCAAGCGCCGAGTCCAATGTCGAG-3’  5’-CCACCAGCCATGAGATCAGCCAGCGAGTCCAATGTCG-3’  5’-GATACCACCAGCCATGAGAGCAAGCAGCGAGTCCAATG-3’  5’-AGGATACCACCAGCCATGGCATCAAGCAGCGAGTCC-3’  5’-CAAAGGATACCACCAGCCGCGAGATCAAGCAGCGAG-3’  5’-ACCAAAGGATACCACCAGTCATGAGATCAAGCAGC-3’  5’-GAACCAAAGGATACCAGCAGCCATGAGATCAAG-3’  5’-GTGTGAACCAAAGGATAGCACCAGCCATGAGATC-3’  5’-AGATGTGTGAACCAAAGGGCACCACCAGCCATGAG-3’  5’-GAGAGATGTGTGAACCAAGCGATACCACCAGCCATG-3’  5’-CTTCATCGAGAGATGTGTGGCCCAAAGGATACCACCAG-3’  5’-CTCTTCATCGAGAGATGTGCGAACCAAAGGATACCAC-3’  5’-ATGCTCTTCATCGAGAGAGCTGTGAACCAAAGGATAC-3’  5’-GATGCTCTTCATCGAGGCATGTGTGAACCAAAG-3’  5’-ACTTGTAGACGTTGATGCCCTTCATCGAGAGATGT-3’  5’-ATACTTGTAGACGTTGGCGCTCTTCATCGAGAGAT-3’  5’-AGGATACTTGTAGACGGCGATGCTCTTCATCGAG-3’  5’-CAATAGGATACTTGTAGGCGTTGATGCTCTTCATCG-3’  5’-ACCAATAGGATACTTGGCGACGTTGATGCTCTTC -3’  5’-GTTTACCAATAGGATACGCGTAGACGTTGATGCTCT-3’  5’-CTCAGTTTACCAATAGGAGCCTTGTAGACGTTGATG-3’  5’-ACCCTCAGTTTACCAATAGCATACTTGTAGACGTTG-3’ |

|  | Sequence of forward primer | Sequence of reverse primer |
| --- | --- | --- |
| I163A  G164A  K165A  R167A  Q169A  V171A  G172A  I173A  I174A  I175A  F176A  A177R  V179A  M180A  T182A  G184A  F185A  Q186A  V187A  F188A  V189A  Q190A  V192A  V207A  T210A | 5’-GTCTACAAGTATCCTGCTGGTAAACTGAGGGTG-3’  5’-CTACAAGTATCCTATTGCTAAACTGAGGGTGCAGCCT-3’  5’-ACAAGTATCCTATTGGTGCACTGAGGGTGCAGCCTGT-3’  5’-ATCCTATTGGTAAACTGGCGGTGCAGCCTGTTGGAAT-3’  5’-ATTGGTAAACTGAGGGTGGCGCCTGTTGGAATCATCAT-3’  5’-ACTGAGGGTGCAGCCTGCTGGAATCATCATCTTTG-3’  5’-GAGGGTGCAGCCTGTTGCAATCATCATCTTTGCTGCT-3’  5’-GTGCAGCCTGTTGGAGCCATCATCTTTGCTGCT-3’  5’-CAGCCTGTTGGAATCGCCATCTTTGCTGCTGTAAT-3’  5’-AGCCTGTTGGAATCATCGCCTTTGCTGCTGTAATGGCT-3’  5’-CTGTTGGAATCATCATCGCTGCTGCTGTAATGGCTAC-3’  5’-GTTGGAATCATCATCTTTCGTGCTGTAATGGCTACAT-3’  5’-ATCATCTTTGCTGCTGCAATGGCTACATTAGGAT-3’  5’-ATCATCTTTGCTGCTGTAGCGGCTACATTAGGATTCC-3’  5’-GCTGCTGTAATGGCTGCATTAGGATTCCAAGTAT-3’  5’-CTGTAATGGCTACATTAGCATTCCAAGTATTTGTTC-3’  5’-ATGGCTACATTAGGAGCCCAAGTATTTGTTCAAG-3’  5’-GCTACATTAGGATTCGCAGTATTTGTTCAAGCTG-3’  5’-ACATTAGGATTCCAAGCATTTGTTCAAGCTGTGG-3’  5’-ATTAGGATTCCAAGTAGCTGTTCAAGCTGTGGAAAAG-3’  5’-AGGATTCCAAGTATTTGCTCAAGCTGTGGAAAAGCTG-3’  5’-ATTCCAAGTATTTGTTGCAGCTGTGGAAAAGCTGAT-3’  5’-AGTATTTGTTCAAGCTGCGGAAAAGCTGATAGTG-3’  5’-AGATAAATTGACTCCAGCGCAGCTCACATGGCTAT-3’  5’-GACTCCAGTGCAGCTCGCATGGCTATATTCTATCAT-3’ | 5’-CACCCTCAGTTTACCAGCAGGATACTTGTAGAC-3’  5’-AGGCTGCACCCTCAGTTTAGCAATAGGATACTTGTAG-3’  5’-ACAGGCTGCACCCTCAGTGCACCAATAGGATACTTGT-3’  5’-ATTCCAACAGGCTGCACCGCCAGTTTACCAATAGGAT-3’  5’-ATGATGATTCCAACAGGCGCCACCCTCAGTTTACCAAT-3’  5’-CAAAGATGATGATTCCAGCAGGCTGCACCCTCAGT-3’  5’-AGCAGCAAAGATGATGATTGCAACAGGCTGCACCCTC-3’  5’-AGCAGCAAAGATGATGGCTCCAACAGGCTGCAC-3’  5’-ATTACAGCAGCAAAGATGGCGATTCCAACAGGCTG-3’  5’-AGCCATTACAGCAGCAAAGGCGATGATTCCAACAGGCT-3’  5’-GTAGCCATTACAGCAGCAGCGATGATGATTCCAACAG-3’  5’-ATGTAGCCATTACAGCACGAAAGATGATGATTCCAAC-3’  5’-ATCCTAATGTAGCCATTGCAGCAGCAAAGATGAT-3’  5’-GGAATCCTAATGTAGCCGCTACAGCAGCAAAGATGAT-3’  5’-ATACTTGGAATCCTAATGCAGCCATTACAGCAGC-3’  5’-GAACAAATACTTGGAATGCTAATGTAGCCATTACAG-3’  5’-CTTGAACAAATACTTGGGCTCCTAATGTAGCCAT-3’  5’-CAGCTTGAACAAATACTGCGAATCCTAATGTAGC-3’  5’-CCACAGCTTGAACAAATGCTTGGAATCCTAATGT-3’  5’-CTTTTCCACAGCTTGAACAGCTACTTGGAATCCTAAT-3’  5’-CAGCTTTTCCACAGCTTGAGCAAATACTTGGAATCCT-3’  5’-ATCAGCTTTTCCACAGCTGCAACAAATACTTGGAAT-3’  5’-CACTATCAGCTTTTCCGCAGCTTGAACAAATACT-3’  5’-ATAGCCATGTGAGCTGCGCTGGAGTCAATTTATCT-3’  5’-ATGATAGAATATAGCCATGCGAGCTGCACTGGAGTC-3’ |

|  | Sequence of forward primer | Sequence of reverse primer |
| --- | --- | --- |
| W211A  L212A  Y213A  S214A  I215A  M216A  I217A  F218A  V222A  K223A  L224A  L226A  W227A  L228A  Y229A  N235A  I237A  V238A  Y241A  D244A  H245A  F247A  D248A  V250A  T251A | 5’-CTCCAGTGCAGCTCACAGCGCTATATTCTATCATGAT-3’  5’-AGTGCAGCTCACATGGGCATATTCTATCATGATTTTTG-3’  5’-CAGCTCACATGGCTAGCTTCTATCATGATTTTTG-3’  5’-GCTCACATGGCTATATGCTATCATGATTTTTGCAAC-3’  5’-CACATGGCTATATTCTGCCATGATTTTTGCAACAG-3’  5’-ATGGCTATATTCTATCGCGATTTTTGCAACAGTAGT-3’  5’-CTATATTCTATCATGGCTTTTGCAACAGTAGTT-3’  5’-ATATTCTATCATGATTGCTGCAACAGTAGTTAAGC-3’  5’-GATTTTTGCAACAGTAGCTAAGCTAGCCCTCTGGCT-3’  5’-ATTTTTGCAACAGTAGTTGCGCTAGCCCTCTGGCTCTAC-3’  5’-GCAACAGTAGTTAAGGCAGCCCTCTGGCTCTAC-3’  5’-CAGTAGTTAAGCTAGCCGCCTGGCTCTACTGCAGAAC-3’  5’-AGTTAAGCTAGCCCTCGCGCTCTACTGCAGAACAT-3’  5’-AAGCTAGCCCTCTGGGCCTACTGCAGAACATCTG-3’  5’-GCTAGCCCTCTGGCTCGCCTGCAGAACATCTGGTAAC-3’  5’-CTGCAGAACATCTGGTGCCAAGATAGTCCGTGCCT-3’  5’-AACATCTGGTAACAAGGCAGTCCGTGCCTATGCTAAG-3’  5’-ATCTGGTAACAAGATAGCCCGTGCCTATGCTAAGG-3’  5’-CAAGATAGTCCGTGCCGCTGCTAAGGATCATTAT-3’  5’-GTCCGTGCCTATGCTAAGGCTCATTATTTTGACGTCGT-3’  5’-GTGCCTATGCTAAGGATGCTTATTTTGACGTCGTCAC-3 ’  5’-ATGCTAAGGATCATTATGCTGACGTCGTCACGAATGT-3’  5’-GCTAAGGATCATTATTTTGCCGTCGTCACGAATGTTGT-3’  5’-ATCATTATTTTGACGTCGCCACGAATGTTGTGGGT-3’  5’-ATTATTTTGACGTCGTCGCGAATGTTGTGGGTTTGGC-3’ | 5’-ATCATGATAGAATATAGCGCTGTGAGCTGCACTGGAG-3’  5’-CAAAAATCATGATAGAATATGCCCATGTGAGCTGCACT-3’  5’-CAAAAATCATGATAGAAGCTAGCCATGTGAGCTG-3’  5’-GTTGCAAAAATCATGATAGCATATAGCCATGTGAGC-3’  5’-CTGTTGCAAAAATCATGGCAGAATATAGCCATGTG-3’  5’-ACTACTGTTGCAAAAATCGCGATAGAATATAGCCAT-3’  5’-AACTACTGTTGCAAAAGCCATGATAGAATATAG-3’  5’-GCTTAACTACTGTTGCAGCAATCATGATAGAATAT-3’  5’-AGCCAGAGGGCTAGCTTAGCTACTGTTGCAAAAATC-3’  5’-GTAGAGCCAGAGGGCTAGCGCAACTACTGTTGCAAAAAT-3’  5’-GTAGAGCCAGAGGGCTGCCTTAACTACTGTTGC-3’  5’-GTTCTGCAGTAGAGCCAGGCGGCTAGCTTAACTACTG-3’  5’-ATGTTCTGCAGTAGAGCGCGAGGGCTAGCTTAACT-3’  5’-CAGATGTTCTGCAGTAGGCCCAGAGGGCTAGCTT-3’  5’-GTTACCAGATGTTCTGCAGGCGAGCCAGAGGGCTAGC-3’  5’-AGGCACGGACTATCTTGGCACCAGATGTTCTGCAG-3’  5’-CTTAGCATAGGCACGGACTGCCTTGTTACCAGATGTT-3’  5’-CCTTAGCATAGGCACGGGCTATCTTGTTACCAGAT-3’  5’-ATAATGATCCTTAGCAGCGGCACGGACTATCTTG-3’  5’-ACGACGTCAAAATAATGAGCCTTAGCATAGGCACGGAC-3’  5’-GTGACGACGTCAAAATAAGCATCCTTAGCATAGGCAC-3’  5’-ACATTCGTGACGACGTCAGCATAATGATCCTTAGCAT-3’  5’-ACAACATTCGTGACGACGGCAAAATAATGATCCTTAGC-3’  5’-ACCCACAACATTCGTGGCGACGTCAAAATAATGAT-3’  5’-GCCAAACCCACAACATTCGCGACGACGTCAAAATAAT-3’ |

|  | Sequence of forward primer | Sequence of reverse primer |
| --- | --- | --- |
| N252A  V253A  G255A  L256A  V260A  L261A  G262A  D263A  M264A  F265A  Y266A  I269A  D270A  P271A  V272A  G273A  A274T  I275A  L277A  V279A  Y280A  T281A  T283A  W285A  G287A | 5’-ATTTTGACGTCGTCACGGCTGTTGTGGGTTTGGCTG-3’  5’-GACGTCGTCACGAATGCTGTGGGTTTGGCTGCT-3’  5’-CGTCACGAATGTTGTGGCTTTGGCTGCTGCTGTCCT-3’  5’-CACGAATGTTGTGGGTGCGGCTGCTGCTGTCCTTG-3’  5’-GTGGGTTTGGCTGCTGCTGCCCTTGGTGATATGTTCT-3’  5’-GTTTGGCTGCTGCTGTCGCTGGTGATATGTTCTACTG-3’  5’-GGCTGCTGCTGTCCTTGCTGATATGTTCTACTGGTG-3’  5’-CTGCTGCTGTCCTTGGTGCTATGTTCTACTGGTGGAT-3’  5’-CTGCTGTCCTTGGTGATGCGTTCTACTGGTGGATTGAC-3’  5’-CTGTCCTTGGTGATATGGCCTACTGGTGGATTGACC-3’  5’-CTTGGTGATATGTTCGCCTGGTGGATTGACCCAG-3’  5’-GATATGTTCTACTGGTGGGCTGACCCAGTTGGTGCAAT-3’  5’-GTTCTACTGGTGGATTGCCCCAGTTGGTGCAATTG-3’  5’-CTACTGGTGGATTGACGCAGTTGGTGCAATTGCCCT-3’  5’-CTGGTGGATTGACCCAGCTGGTGCAATTGCCCTTG-3’  5’-GTGGATTGACCCAGTTGCTGCAATTGCCCTTGCAG-3’  5’-GATTGACCCAGTTGGTACAATTGCCCTTGCAGTGT-3’  5’-GACCCAGTTGGTGCAGCTGCCCTTGCAGTGTACAC-3’  5’-AGTTGGTGCAATTGCCGCTGCAGTGTACACAATTAC-3’  5’-GTGCAATTGCCCTTGCAGCGTACACAATTACAAACTG-3’  5’-CAATTGCCCTTGCAGTGGCCACAATTACAAACTGGTC-3’  5’-ATTGCCCTTGCAGTGTACGCAATTACAAACTGGTCTG-3’  5’-GCAGTGTACACAATTGCAAACTGGTCTGGAACGGT-3’  5’-GTACACAATTACAAACGCGTCTGGAACGGTGTGG-3’  5’-CAATTACAAACTGGTCTGCAACGGTGTGGGAAAATG-3’ | 5’-CAGCCAAACCCACAACAGCCGTGACGACGTCAAAAT-3’  5’-AGCAGCCAAACCCACAGCATTCGTGACGACGTC-3’  5’-AGGACAGCAGCAGCCAAAGCCACAACATTCGTGACG-3’  5’-CAAGGACAGCAGCAGCCGCACCCACAACATTCGTG-3’  5’-AGAACATATCACCAAGGGCAGCAGCAGCCAAACCCAC-3’  5’-CAGTAGAACATATCACCAGCGACAGCAGCAGCCAAAC-3’  5’-CACCAGTAGAACATATCAGCAAGGACAGCAGCAGCC-3’  5’-ATCCACCAGTAGAACATAGCACCAAGGACAGCAGCAG-3’  5’-GTCAATCCACCAGTAGAACGCATCACCAAGGACAGCAG-3’  5’-GGTCAATCCACCAGTAGGCCATATCACCAAGGACAG-3’  5’-CTGGGTCAATCCACCAGGCGAACATATCACCAAG-3’  5’-ATTGCACCAACTGGGTCAGCCCACCAGTAGAACATATC-3’  5’-CAATTGCACCAACTGGGGCAATCCACCAGTAGAAC-3’  5’-AGGGCAATTGCACCAACTGCGTCAATCCACCAGTAG-3’  5’-CAAGGGCAATTGCACCAGCTGGGTCAATCCACCAG-3’  5’-CTGCAAGGGCAATTGCAGCAACTGGGTCAATCCAC-3’  5’-ACACTGCAAGGGCAATTGTACCAACTGGGTCAATC-3’  5’-GTGTACACTGCAAGGGCAGCTGCACCAACTGGGTC-3’  5’-GTAATTGTGTACACTGCAGCGGCAATTGCACCAACT-3’  5’-CAGTTTGTAATTGTGTACGCTGCAAGGGCAATTGCAC-3’  5’-GACCAGTTTGTAATTGTGGCCACTGCAAGGGCAATTG-3’  5’-CAGACCAGTTTGTAATTGCGTACACTGCAAGGGCAAT-3’  5’-ACCGTTCCAGACCAGTTTGCAATTGTGTACACTGC-3’  5’-CCACACCGTTCCAGACGCGTTTGTAATTGTGTAC-3’  5’-CATTTTCCCACACCGTTGCAGACCAGTTTGTAATTG-3’ |

|  | Sequence of forward primer | Sequence of reverse primer |
| --- | --- | --- |
| T288A  W290A  N292A  A293T  V294A  L296A  G298A  E299A  S300A  P302A  P303A  E304A  L306A  Q307A  K308A  L312A  I314A  H316A  I320A  K321A  D324A  T325A  V326A  R327A  A328T | 5’-ATTACAAACTGGTCTGGAGCGGTGTGGGAAAATGCAG-3’  5’-ACTGGTCTGGAACGGTGGCGGAAAATGCAGTGTCAC-3’  5’-CTGGAACGGTGTGGGAAGCTGCAGTGTCACTAGTGG-3  5’-GAACGGTGTGGGAAAATACAGTGTCACTAGTGGGTG-3’  5’-CGGTGTGGGAAAATGCAGCGTCACTAGTGGGTGAAT-3’  5’-GGAAAATGCAGTGTCAGCAGTGGGTGAATCAGCTC-3’  5’-ATGCAGTGTCACTAGTGGCTGAATCAGCTCCGCCAG-3’  5’-CAGTGTCACTAGTGGGTGCATCAGCTCCGCCAGAAAT-3’  5’-GTCACTAGTGGGTGAAGCAGCTCCGCCAGAAATGCT-3’  5’-AGTGGGTGAATCAGCTGCGCCAGAAATGCTGCAG-3’  5’-GTGGGTGAATCAGCTCCGGCAGAAATGCTGCAGAAG-3’  5’-GTGAATCAGCTCCGCCAGCAATGCTGCAGAAGTTGAC-3’  5’-CAGCTCCGCCAGAAATGGCGCAGAAGTTGACATAT-3’  5’-CTCCGCCAGAAATGCTGGCGAAGTTGACATATTTGG-3’  5’-GCCAGAAATGCTGCAGGCGTTGACATATTTGGCCAT-3’  5’-GCAGAAGTTGACATATGCGGCCATCAGACATCATC-3’  5’-AGTTGACATATTTGGCCGCCAGACATCATCCTCAAAT-3’  5’-CATATTTGGCCATCAGAGCTCATCCTCAAATCAAGC-3’  5’CAGACATCATCCTCAAGCCAAGCGTGTCGATACAG-3’  5’-ACATCATCCTCAAATCGCGCGTGTCGATACAGTTC-3’  5’-CTCAAATCAAGCGTGTCGCTACAGTTCGAGCATACAC-3’  5’-CAAATCAAGCGTGTCGATGCAGTTCGAGCATACACC-3’  5’-CAAGCGTGTCGATACAGCTCGAGCATACACCTTTG-3’  5’-GCGTGTCGATACAGTTGCAGCATACACCTTTGGGGT-3’  5’-GTGTCGATACAGTTCGAACATACACCTTTGGGGTTC-3 | 5’-CTGCATTTTCCCACACCGCTCCAGACCAGTTTGTAAT-3’  5’-GTGACACTGCATTTTCCGCCACCGTTCCAGACCAGT-3’  5’-CCACTAGTGACACTGCAGCTTCCCACACCGTTCCAG-3’  5’-CACCCACTAGTGACACTGTATTTTCCCACACCGTTC-3’  5’-ATTCACCCACTAGTGACGCTGCATTTTCCCACACCG-3’  5’-GAGCTGATTCACCCACTGCTGACACTGCATTTTCC-3’  5’-CTGGCGGAGCTGATTCAGCCACTAGTGACACTGCAT-3’  5’-ATTTCTGGCGGAGCTGATGCACCCACTAGTGACACTG-3’  5’-AGCATTTCTGGCGGAGCTGCTTCACCCACTAGTGAC-3’  5’-CTGCAGCATTTCTGGCGCAGCTGATTCACCCACT-3’  5’-CTTCTGCAGCATTTCTGCCGGAGCTGATTCACCCAC-3’  5’-GTCAACTTCTGCAGCATTGCTGGCGGAGCTGATTCAC-3’  5’-ATATGTCAACTTCTGCGCCATTTCTGGCGGAGCTG-3’  5’-CCAAATATGTCAACTTCGCCAGCATTTCTGGCGGAG-3’  5’-ATGGCCAAATATGTCAACGCCTGCAGCATTTCTGGC-3’  5’-GATGATGTCTGATGGCCGCATATGTCAACTTCTGC-3’  5’-ATTTGAGGATGATGTCTGGCGGCCAAATATGTCAACT-3’  5’-GCTTGATTTGAGGATGAGCTCTGATGGCCAAATATG-3’  5’-CTGTATCGACACGCTTGGCTTGAGGATGATGTCTG-3’  5’-GAACTGTATCGACACGCGCGATTTGAGGATGATGT-3’  5’-GTGTATGCTCGAACTGTAGCGACACGCTTGATTTGAG-3’  5’-GGTGTATGCTCGAACTGCATCGACACGCTTGATTTG-3’  5’-CAAAGGTGTATGCTCGAGCTGTATCGACACGCTTG-3’  5’-ACCCCAAAGGTGTATGCTGCAACTGTATCGACACGC-3’  5’-GAACCCCAAAGGTGTATGTTCGAACTGTATCGACAC-3’ |

|  | Sequence of forward primer | Sequence of reverse primer |
| --- | --- | --- |
| Y329A  G332A  V333A  L334A  E338A  V339A  I341A  E342A  L343A  E345A  L347A  L349A  E351A  H353A  A354T  I355A  E357A  S358A  L359A  Q360A  I361A  K362A  I363A  E364A  E365A | 5’-CGATACAGTTCGAGCAGCCACCTTTGGGGTTCTTTAC-3’  5’-CGAGCATACACCTTTGCGGTTCTTTACTTTGTTG-3’  5’-AGCATACACCTTTGGGGCTCTTTACTTTGTTGAGGT-3’  5’-ATACACCTTTGGGGTTGCTTACTTTGTTGAGGTTG-3’  5’-GTTCTTTACTTTGTTGCGGTTGACATTGAGCTGC-3’  5’-CTTTACTTTGTTGAGGCTGACATTGAGCTGCCAG-3’  5’-ACTTTGTTGAGGTTGACGCTGAGCTGCCAGAGGAAT-3’  5’-GTTGAGGTTGACATTGCGCTGCCAGAGGAATTGC-3’  5’-GAGGTTGACATTGAGGCGCCAGAGGAATTGCCCCT-3’  5’-GACATTGAGCTGCCAGCGGAATTGCCCCTTAAGGAG-3’  5’-GAGCTGCCAGAGGAAGCGCCCCTTAAGGAGGCAC-3’  5’-GCCAGAGGAATTGCCCGCTAAGGAGGCACATGCTAT-3’  5’-GAATTGCCCCTTAAGGCGGCACATGCTATTGGAG-3’  5’-TGCCCCTTAAGGAGGCAGCTGCTATTGGAGAATCACT-3’  5’-CTTAAGGAGGCACATACTATTGGAGAATCACTTC -3’  5’-TAAGGAGGCACATGCTGCTGGAGAATCACTTCAAAT-3’  5’-AGGCACATGCTATTGGAGCATCACTTCAAATAAAGAT-3’  5’-ACATGCTATTGGAGAAGCACTTCAAATAAAGATC-3  5’-ATGCTATTGGAGAATCAGCTCAAATAAAGATCGAGG-3’  5’-CTATTGGAGAATCACTTGCAATAAAGATCGAGGAGC-3’  5’-GGAGAATCACTTCAAGCAAAGATCGAGGAGCTCC-3’  5’-AGAATCACTTCAAATAGCGATCGAGGAGCTCCCAG-3’  5’-ATCACTTCAAATAAAGGCCGAGGAGCTCCCAGAAG-3’  5’-ACTTCAAATAAAGATCGCGGAGCTCCCAGAAGTCG-3’  5’-CAAATAAAGATCGAGGCGCTCCCAGAAGTCGAGC-3’ | 5’-GTAAAGAACCCCAAAGGTGGCTGCTCGAACTGTATCG-3’  5’-CAACAAAGTAAAGAACCGCAAAGGTGTATGCTCG-3’  5’-ACCTCAACAAAGTAAAGAGCCCCAAAGGTGTATGCT-3’  5’-CAACCTCAACAAAGTAAGCAACCCCAAAGGTGTAT-3’  5’-GCAGCTCAATGTCAACCGCAACAAAGTAAAGAAC-3’  5’-CTGGCAGCTCAATGTCAGCCTCAACAAAGTAAAG-3’  5’-ATTCCTCTGGCAGCTCAGCGTCAACCTCAACAAAGT-3’  5’-GCAATTCCTCTGGCAGCGCAATGTCAACCTCAAC -3’  5’-AGGGGCAATTCCTCTGGCGCCTCAATGTCAACCTC-3’  5’-CTCCTTAAGGGGCAATTCCGCTGGCAGCTCAATGTC-3’  5’-GTGCCTCCTTAAGGGGCGCTTCCTCTGGCAGCTC-3’  5’-ATAGCATGTGCCTCCTTAGCGGGCAATTCCTCTGGC-3’  5’-CTCCAATAGCATGTGCCGCCTTAAGGGGCAATTC-3’  5’-AGTGATTCTCCAATAGCAGCTGCCTCCTTAAGGGGCA-3  5’-GAAGTGATTCTCCAATAGTATGTGCCTCCTTAAG-3’  5’-ATTTGAAGTGATTCTCCAGCAGCATGTGCCTCCTTA-3’  5’-ATCTTTATTTGAAGTGATGCTCCAATAGCATGTGCCT-3’  5’-GATCTTTATTTGAAGTGCTTCTCCAATAGCATGT-3’  5’-CCTCGATCTTTATTTGAGCTGATTCTCCAATAGCAT-3’  5’-GCTCCTCGATCTTTATTGCAAGTGATTCTCCAATAG-3’  5’-GGAGCTCCTCGATCTTTGCTTGAAGTGATTCTCC-3’  5’-CTGGGAGCTCCTCGATCGCTATTTGAAGTGATTCT-3’  5’-CTTCTGGGAGCTCCTCGGCCTTTATTTGAAGTGAT-3’  5’-CGACTTCTGGGAGCTCCGCGATCTTTATTTGAAGT-3’  5’-GCTCGACTTCTGGGAGCGCCTCGATCTTTATTTG-3’ |

|  | Sequence of forward primer | Sequence of reverse primer |
| --- | --- | --- |
| E368A  E370A  R371A  A372T  L376A  D377A  E379A  C380A  K383A  E385A  H386A  N387A  I388A  L389A  S390A  K391A  △2-29  △2-55  △2-60  △2-79  △2-85  △2-90  △2-99  △297-396  △302-396 | 5’-GATCGAGGAGCTCCCAGCAGTCGAGCGGGCATTTG-3’  5’-GGAGCTCCCAGAAGTCGCGCGGGCATTTGTTCACCT-3’  5’-AGCTCCCAGAAGTCGAGGCGGCATTTGTTCACCTTG-3’  5’-CCAGAAGTCGAGCGGACATTTGTTCACCTTGATT-3’  5’-AGCGGGCATTTGTTCACGCTGATTTCGAGTGTGACC-3’  5’-GGCATTTGTTCACCTTGCTTTCGAGTGTGACCACAAG-3’  5’-GTTCACCTTGATTTCGCGTGTGACCACAAGCCAG-3’  5’-CACCTTGATTTCGAGGCTGACCACAAGCCAGAGC-3’  5’-ATTTCGAGTGTGACCACGCGCCAGAGCACAACATTC-3’  5’-GTGTGACCACAAGCCAGCGCACAACATTCTCAGCAAG-3’  5’-GTGACCACAAGCCAGAGGCCAACATTCTCAGCAAGTT-3’  5’-ACCACAAGCCAGAGCACGCCATTCTCAGCAAGTTGC-3’  5’-ACAAGCCAGAGCACAACGCTCTCAGCAAGTTGCCTAG-3’  5’-AGCCAGAGCACAACATTGCCAGCAAGTTGCCTAGCAG-3’  5’-AGAGCACAACATTCTCGCCAAGTTGCCTAGCAGCC-3’  5’-AGCACAACATTCTCAGCGCGTTGCCTAGCAGCCAAC-3’  5’-CAAATATAAAACCAGCGGCCGCATGGTGTCGCGGCTGCCCAAGAAG-3’  5’-CAAATATAAAACCAGCGGCCGCATGGGCCTCCGCGAAGGAGAGAAAG-3’  5’-CAAATATAAAACCAGCGGCCGCATGGAGAAAGAATACTATGAGAAAC-3’  5’-CAAATATAAAACCAGCGGCCGCATGTCAATAGAAGAATCCAATGTAATGAG-3’  5’-CAAATATAAAACCAGCGGCCGCATGGTAATGAGTGAAGAAGATGAT-3’  5’-CAAATATAAAACCAGCGGCCGCATGGATGATATTGCGGAGCAAAAG -3’  5’-CAAATATAAAACCAGCGGCCGCATGGAGTTTGCCATGAAGATATC-3’  5’-CAAATATAAAACCAGCGGCCGCATGGAGGCGAAGGGGGAGAATGAC-3’ 5’-CAAATATAAAACCAGCGGCCGCATGGAGGCGAAGGGGGAGAATGAC-3’ | 5’-CAAATGCCCGCTCGACTGCTGGGAGCTCCTCGATC-3’  5’-AGGTGAACAAATGCCCGCGCGACTTCTGGGAGCTCC-3’  5’-CAAGGTGAACAAATGCCGCCTCGACTTCTGGGAGCT-3’  5’-AATCAAGGTGAACAAATGTCCGCTCGACTTCTGG-3’  5’-GGTCACACTCGAAATCAGCGTGAACAAATGCCCGCT-3’  5’-CTTGTGGTCACACTCGAAAGCAAGGTGAACAAATGCC-3’  5’-CTGGCTTGTGGTCACACGCGAAATCAAGGTGAAC-3’  5’-GCTCTGGCTTGTGGTCAGCCTCGAAATCAAGGTG-3’  5’-GAATGTTGTGCTCTGGCGCGTGGTCACACTCGAAAT-3’  5’-CTTGCTGAGAATGTTGTGCGCTGGCTTGTGGTCACAC-3’  5’-AACTTGCTGAGAATGTTGGCCTCTGGCTTGTGGTCAC-3’  5’-CTAGGCAACTTGCTGAGAGCGTTGTGCTCTGGCTTGT-3’  5’-GCAACTTGCTGAGAATGGCGTGCTCTGGCTTGTGGT-3’  5’-CTGCTAGGCAACTTGCTGGCAATGTTGTGCTCTGGCT-3’  5’-GGCTGCTAGGCAACTTGGCGAGAATGTTGTGCTCT-3’  5’-GTTGGCTGCTAGGCAACGCGCTGAGAATGTTGTGCT-3’  5’-GAAAAAAATTGATCGCGGCCGCTCATGGTTGGCTGCTAGGCAACTTG-3’  5’-GAAAAAAATTGATCGCGGCCGCTCATGGTTGGCTGCTAGGCAACTTG-3’  5’-GAAAAAAATTGATCGCGGCCGCTCATGGTTGGCTGCTAGGCAACTTG-3’  5’-GAAAAAAATTGATCGCGGCCGCTCATGGTTGGCTGCTAGGCAACTTG-3’  5’-GAAAAAAATTGATCGCGGCCGCTCATGGTTGGCTGCTAGGCAACTTG-3’  5’-GAAAAAAATTGATCGCGGCCGCTCATGGTTGGCTGCTAGGCAACTTG-3’  5’-GAAAAAAATTGATCGCGGCCGCTCATGGTTGGCTGCTAGGCAACTTG-3’  5’- GAAAAAAATTGATCGCGGCCGCTCATGGCGGAGCTGATTCACCCAC-3’  5’-GAAAAAAATTGATCGCGGCCGCTCAAGCTGATTCACCCACTAGTGACA-3’ |

|  | Sequence of forward primer | Sequence of reverse primer |
| --- | --- | --- |
| △334-396  △371-396  △378-396  △385-396  △392-396 | 5’-CAAATATAAAACCAGCGGCCGCATGGAGGCGAAGGGGGAGAATGAC-3’  5’-CAAATATAAAACCAGCGGCCGCATGGAGGCGAAGGGGGAGAATGAC-3’  5’-CAAATATAAAACCAGCGGCCGCATGGAGGCGAAGGGGGAGAATGAC-3’  5’-CAAATATAAAACCAGCGGCCGCATGGAGGCGAAGGGGGAGAATGAC-3’  5’-CAAATATAAAACCAGCGGCCGCATGGAGGCGAAGGGGGAGAATGAC-3’ | 5’-GAAAAAAATTGATCGCGGCCGCTCAAACCCCAAAGGTGTATGCTCGAA-3’  5’-GAAAAAAATTGATCGCGGCCGCTCACTCGACTTCTGGGAGCTCCTCGA-3’  5’-GAAAAAAATTGATCGCGGCCGCTCAATCAAGGTGAACAAATGCCCGC-3’  5’-GAAAAAAATTGATCGCGGCCGCTCATGGCTTGTGGTCACACTCGAAAT-3’  5’-GAAAAAAATTGATCGCGGCCGCTCACTTGCTGAGAATGTTGTGCTCTG-3’ |

**Supplemental Figure legends**

**Figure S1. Identification of OsMTP8.1 random mutations that lost the ability to confer manganese tolerance.** Wild-type (BY4741), pmr1, OsMTP8.1 and OsMTP8.1 mutants were grown on SD-Ura plates with 6 mM MnCl_2_. Plates were incubated at 30°C for 3 days.

**Figure S2. Mutations conferred nearly the same level of Mn tolerance as that of wild-type *Os*MTP8.1.** Complementation of *S. cerevisiae* mutant *pmr1* grown on selective media supplemented with 3mM and 8mM Mn^2+^. For complementation tests, transformants were pre-cultured in SD-Urea overnight. Pre-cultured cells were diluted to an OD_600 nm_ of 1.0, 10 μL of cell suspensions were spotted on SD-Urea plates supplemented with the indicated concentrations of Mn^2+^. Plates were incubated for 72 h at 30 °C.

**Figure S3. Two mutants F75A and S132A unable to confer tolerance to other metals including Cd, Ni, Cu, Co or Fe.** Dilution series of wild-type and mutant yeast strains transformed with *Os*MTP8.1, the empty vector or the mutant *Os*MTP8.1 were spotted onto plates supplemented with metals as indicated.

**Figure S4. Vacuolar membrane localization of mutated *Os*MTP8.1.** C-terminal GFP fusion protein expressed in the *S. cerevisiae* strain *Δpmr1*. Empty vector represents the plasmid pFL61-GFP. Cells are visualized 24 h after induction. From left to right, shown are GFP fluorescence, bright-field, and merged images with GFP in green. Scale bar 5μm.

**Figure S5. 3D model of *Os*MTP8.1.** A 3D model of the *Os*MTP8.1 homodimer was constructed by using SWISSMODEL software based on the X-ray crystal structure of *E. coli* Zn^2+^ transporter YiiP (PDB ID 3H90). The N-terminal cytosolic domains of *Os*MTP8.1 were omitted from the model.

**Figure S1.**

**
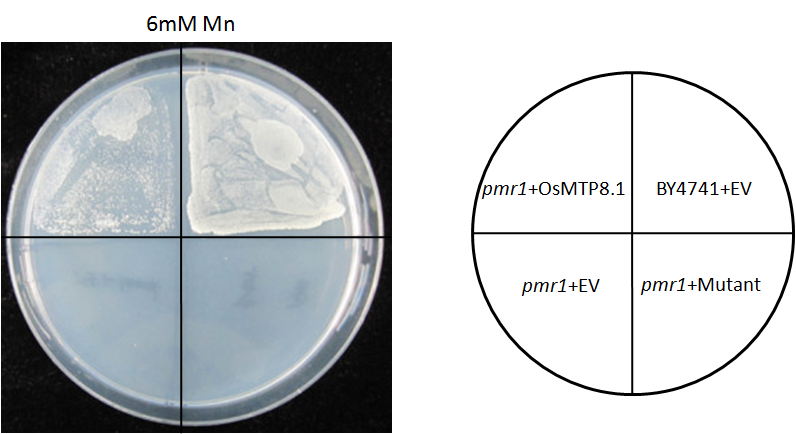
**

**Figure S2.**

**
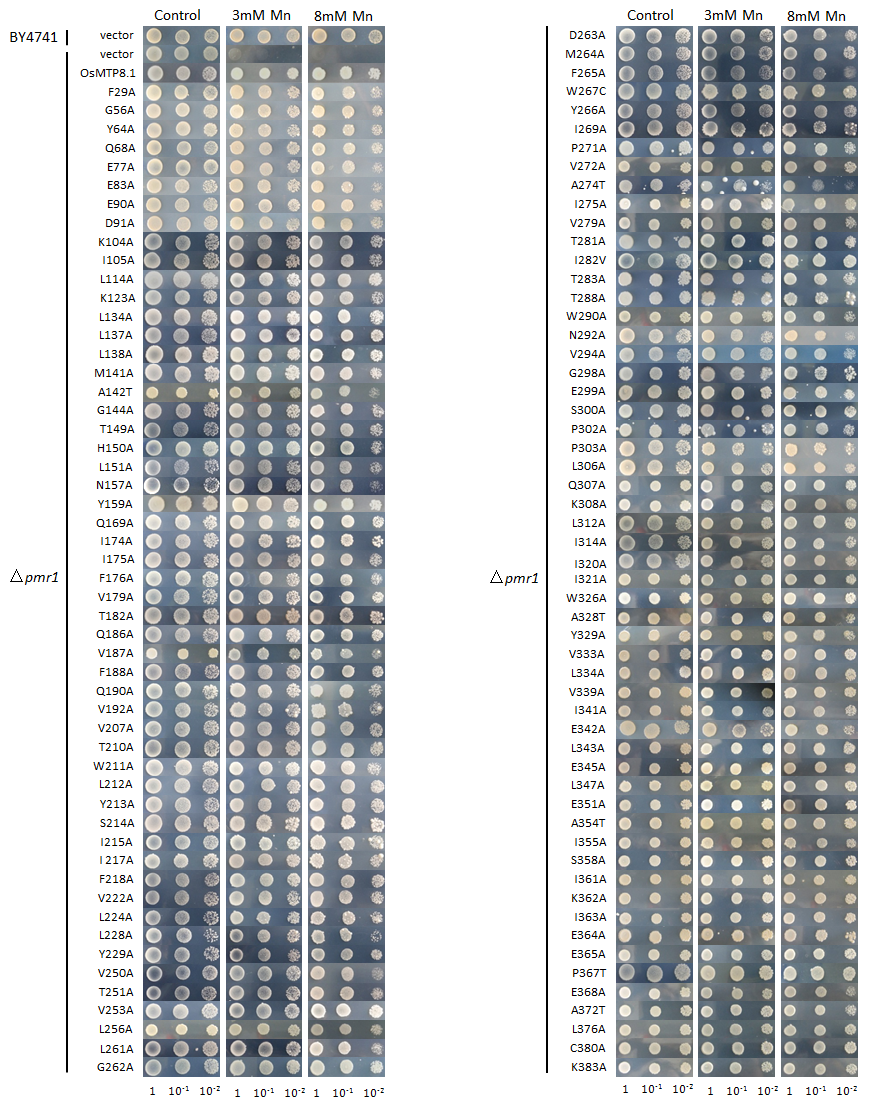
**

**Figure S3.**


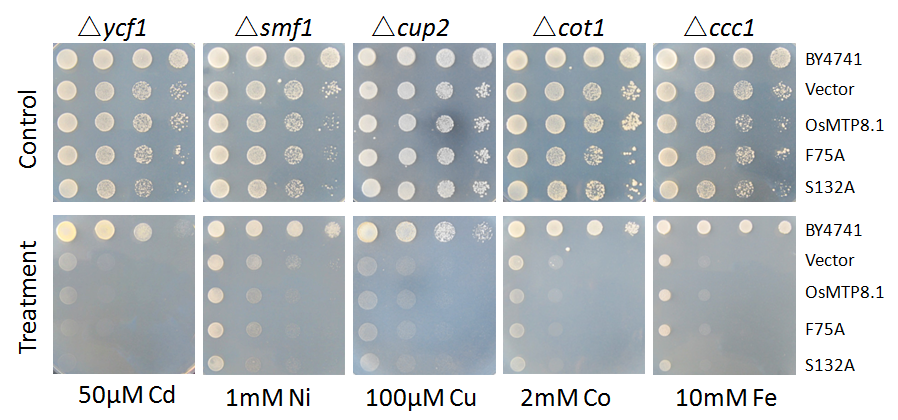


**Figure S4.**

**
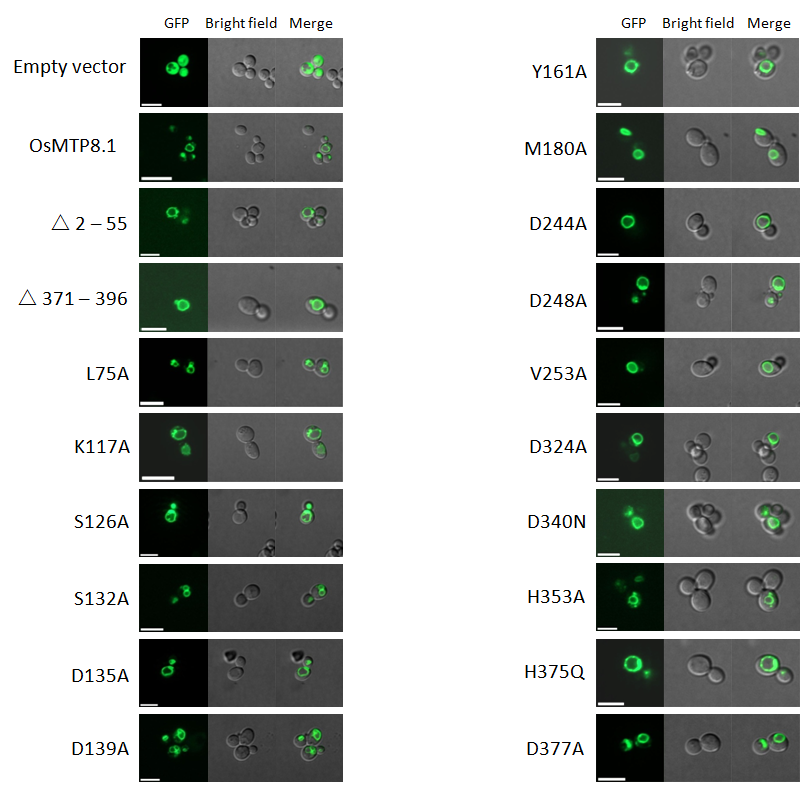
**

**Figure S5.**

**
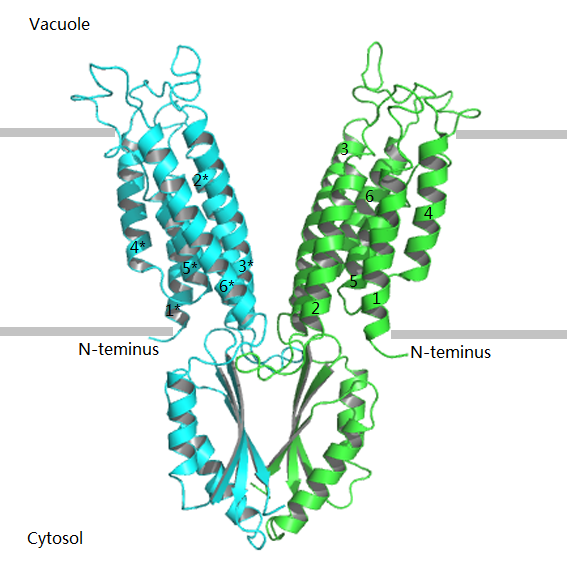
**
